# Efficacy and safety of Osteoking on fracture healing: a systematic review and meta-analysis

**DOI:** 10.3389/fphar.2024.1363421

**Published:** 2024-06-10

**Authors:** Le Zhang, Haomin Kuang, Zimin Zhang, Kuan Rong, Yiwei Yuan, Zhifei Peng, Haomin Zhao, Ke Liu, Liang Ou, Jianjun Kuang

**Affiliations:** ^1^ The Academy of Chinese Medicine, Hunan University of Chinese Medicine, Changsha, China; ^2^ Hunan Academy of Chinese Medicine, Changsha, China; ^3^ Affiliated Hospital of Hunan Academy of Traditional Chinese Medicine, Changsha, China

**Keywords:** Osteoking, fracture healing, randomized controlled trial, meta-analysis, systematic review

## Abstract

**Background:**

Osteoking (OK) is prescribed in traditional Chinese medicine to accelerate fracture healing. Although some studies suggest the potential efficacy of OK for fracture healing, the evidence remains inconclusive.

**Aim:**

To systematically evaluate the safety of OK and its effect on fracture healing.

**Methods:**

Relevant authoritative databases were searched until 25 August 2023. Randomized controlled trials (RCTs) of patients with fractures treated with Osteoking were included. We evaluated the risk of bias using the Cochrane tool and performed a meta-analysis using the Review Manager 5.4 software package.

**Results:**

13 studies involving 1123 participants were included. This meta-analysis showed that compared with observations in the control group, the OK group showed a shortened fracture healing time, increased fracture healing rate, reduced swelling regression time and ecchymosis regression time, and improved bone metabolism. In addition, the included studies did not report any serious side effects associated with the use of OK, and the mild side effects resolved without treatment.

**Conclusion:**

OK therapy is beneficial and safe for accelerating fracture healing, reducing swelling, eliminating ecchymosis, and improving bone metabolism. However, the meta-analysis results do not support OK treatment for improving the fracture healing rate at all fracture sites and reducing pain across all fracture sites. Further original, high-quality studies are needed to validate these findings.

**Systematic Review Registration:** https://www.crd.york.ac.uk/PROSPERO/display_record.php?RecordID=452430, identifier CRD42023452430.

## 1 Introduction

Fracture, a common disease to the musculoskeletal system, which can be divided into two categories based on the cause of the injury: traumatic and non-traumatic (Einhorn and Gerstenfeld, 2015). Despite the considerable regenerative potential of bone, fractures remain at high risk for impaired healing (Wildemann et al., 2021). According to available survey reports, 5%–10% of patients with fracture experience impaired fracture healing worldwide, and the prevalence of non-union varies by site (Tzioupis and Giannoudis, 2007). Fracture healing depends on multiple factors such as severity of injury, chronic disease, age, and malnutrition (Ehnert et al., 2020). Prolonged fracture non-union is a chronic disease that affects daily life, functional recovery and increases financial strain. The average direct cost of treating nonunion in long bones is estimated to be $11,333 in the United States, $11,800 in Canada, and £29,204 in the United Kingdom (Papachristou et al., 2021). Accelerated fracture healing is beneficial in reducing non-union and other fracture complications, such as joint stiffness and muscle atrophy. Currently, although standardized treatments for delayed-healing fractures or non-union exist, including autologous bone grafting, clinically effective methods for promoting fracture healing are lacking to date (Schlundt et al., 2018; Colucci et al., 2021). Furthermore, medications commonly used in the early stages of fractures, such as Nonsteroidal Anti-inflammatory Drugs, may inhibit fracture healing (Zhao-Fleming et al., 2018). Therefore, there is an increasing demand for safe and effective adjuvant treatments to accelerate fracture healing.

Chinese herbs have been utilized for thousands of years to promote fracture healing with few side effects and unique benefits. Osteoking (OK) is a proprietary Chinese medicine developed based on the summary of 600 years of fracture medication experience of the Yi ethnic group in China, which is effective in promoting fracture healing (Yuan et al., 2019). Furthermore, it is registered with the US Food and Drug Administration (#200004068) and is recommended by national and international guidelines for the treatment of fractures, osteoarthritis, and other orthopedic conditions (Tong, 2018; Yu et al., 2019). The details of OK, including the source, composition, dosage, extraction procedure, indications, etc., are showed in Supplementary Material S1.

In recent years, an increasing number of clinical studies have evaluated the effects of OK in the treatment of patients with fractures. However, there is a lack of systematic research evaluating the efficacy and safety of OK in these patients. Therefore, to provide a reference for fracture treatment, this study systematically evaluated the safety and efficacy of OK on fracture healing, pain relief, reduction of swelling and ecchymosis, and bone metabolism.

## 2 Materials and methods

### 2.1 Protocol and registration

This meta-analysis has been registered in the international Prospective Register of Systematic Reviews, number CRD42023452430 (https://www.crd.york.ac.uk/PROSPERO/display_record.php?RecordID=452430). This meta-analysis was performed and reported according to the Preferred Reporting Items for Systematic Reviews and Meta-Analyses (Page et al., 2021).

### 2.2 Search strategy

Potentially eligible trials were searched in PubMed, Embase, Cochrane Library, China National Knowledge Infrastructure (CNKI), Chinese Scientific Journals Database (VIP), Chinese Biomedical Literature Database (CBM) and Wanfang data, up to 25 August 2023. The search strategy used medical subject headings terms in combination with free text, such as “Fracture Healing,” “Bone Fracture,” “Broken Bones,” “Chinese Herbal Drugs,” “Chinese Plant Extracts,” and “Osteoking,” etc. The detailed search strategies are presented in Supplementary Material S2.

### 2.3 Inclusion criteria

#### 2.3.1 Types of studies

RCTs related to the application of OK in fracture healing were searched for. There were no limitations regarding language, publication year, etc.

#### 2.3.2 Types of participants

Patients diagnosed with fracture were included. Fracture was diagnosed by x-ray, computed tomography, and other imaging studies in combination with patient symptoms and physical examination based on a well-defined definition or internationally recognized diagnostic criteria. Any fracture type (fresh, old, open, closed) at any site (irregular, flat, long, or short) was considered acceptable.

#### 2.3.3 Intervention

The same surgical treatment was given to both the control and experimental groups. The experimental group received OK treatment or OK combined with conventional treatment after operation. The control group (CG) received conventional treatment or other traditional Chinese medicine treatments. The ingredients of Osteoking are *Carthamus tinctorius* L., *Panax notoginseng* F.H.Chen*, Eucommia ulmoides* Oliv, *Panax ginseng* C.A.Mey, *Citrus reticulata Blanco* D.C., *Astragalus hamosus* L., *Datura metel* L., *Trionyx sinensis* W. and *Schizophragma integrifolium* Oliv. with 25 mL a bottle.

#### 2.3.4 Outcomes

The primary outcomes included the fracture healing time (FHT) and the fracture healing rate (FHR). The secondary outcomes included the swelling regression time (SRT), ecchymosis regression time (ERT), visual analogue scale (VAS), alkaline phosphatase (ALP), Bone Gla-protein (BGP), and propeptide of type Ⅰprocollagen (PICP).

### 2.4 Exclusion criteria

(1) The experimental group received other therapeutic interventions with Chinese medicine (such as Chinese herbal medicine and electrical acupuncture).
(2) Protocols, reviews, and animal experimental studies.
(3) Duplicate articles and full-text or non-available data papers.
(4) Studies with academic dishonesties, including plagiarism and falsification of data, were excluded.

### 2.5 Literature screening and data extraction

Two investigators used a predesigned spreadsheet to separately extract the essential content from the included papers: lead author, year of publication, patient age and sex, type of intervention, fracture type, dosage, course of treatment, and outcome. Any discrepancies in the crosschecking procedures were resolved through discussions. Otherwise, the dispute was subject to arbitration by a third party or another researcher.

### 2.6 Risk of bias assessment

The methodological quality of all included studies was evaluated independently by two reviewers following the standards recommended in the Cochrane manual (Higgins Jpt, 2021). Discrepancies were resolved by discussion with a third author. Risk of bias for each trial was assessed from seven perspectives: sequence generation, allocation concealment, participant and personnel blinding, outcome assessment blinding, incomplete outcome data, selective reporting, and other bias. There are three levels of risk: high, low, or unclear, based on the evaluation result for each item.

### 2.7 Statistical analysis

Review Manager 5.4 was applied to all meta-analyses of observational indicators in the selected literature, and the corresponding results were intuitively displayed on the forest plot. In this review, we used mean difference (MD) to pool continuous variables. If each original study outcome indicator unit is inconsistent, the Standard Mean Difference (SMD) alternative MD should be selected. And dichotomous variables were pooled using the odds ratio (OR). All pooling effects are reported with 95% confidence intervals (95% CI). A *p*-value of less than 0.05 was considered statistically significant. The test for heterogeneity was performed using the I^2^ statistic and the Cochran Q testing. High heterogeneity was indicated by an I^2^ statistic >50%. Fixed effects model is used for I^2^ statistic <50%, otherwise random effects model is selected. Sensitivity analysis tested the stability of the results. Stata 14 was used to estimate publication bias using Egger’s tests.

### 2.8 Quality of evidence

The quality of evidence for the main outcomes was assessed using the Grading of Recommendations, Assessment, Development and Evaluation (GRADE) approach.

## 3 Results

### 3.1 Results of the search

**FIGURE 1:**
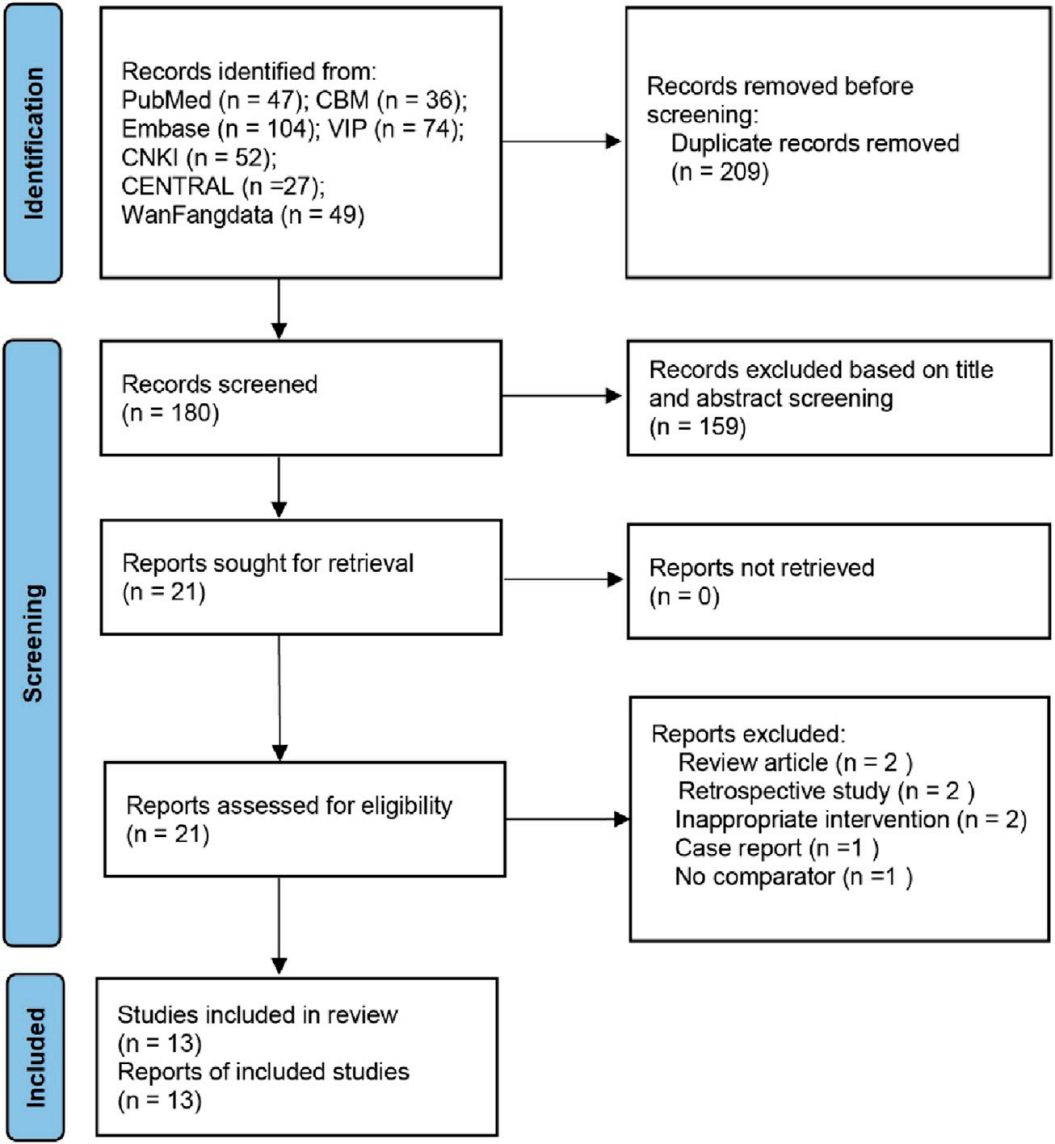
Flow diagram of literature search.

The literature search identified 389 publications; 209 were excluded due to duplication and 159 were excluded by reading the abstracts. After further full-text screening, a total of 13 studies on the effects of OK on fracture healing were included in the analysis based on the screening criteria. A flowchart of the selection process is shown in Figure 1.

### 3.2 Characteristics of studies

A total of 13 studies (Hu et al., 2005; Luo et al., 2011; Zhang et al., 2013; Zhu, 2014; Shen, 2015; He et al., 2020; Han and Liu, 2021; Li et al., 2021; Hao et al., 2022; He et al., 2022; Wang et al., 2023a; Wang et al., 2023b; Min, 2023) were included in this systematic evaluation, with 573 cases in the experimental group and 550 cases in the control group. All studies had small sample sizes, ranging from 24 to 143 participants. In terms of interventions, patients in the experimental group received OK treatment along with basic treatments, such as surgery and pain control. Patients in the CG were treated with conventional surgery, anti-inflammatory and pain-relieving agents, anti-osteoporotic agents, or other Chinese medicines. Characteristics of studies are shown in Table 1. A summary of the composition characteristics of the preparations included in all studies can be found in the Supplementary Material S3.

**TABLE 1:** The characteristics of the included studies.

First author (year)	Age (years)		Gender (Male/Female)		Sample size		Fracture type	Study design	Intervention			Outcomes
	EG	CG	EG	CG	EG	CG			EG	CG	Dosage/Course of treatment	
Min (2023)	74.9 ± 5.8	73.6 ± 5.2	17/19	18/16	36	34	Hip	RCT	Osteoking	Taohong siwu decoction	25mL, qod/4 weeks	FHT, SRT, ERT, ALP, BGP, PICP
Wang et al. (2023a)	38.74 ± 4.98	40.12 ± 4.83	18/10	22/6	28	28	Limbs long backbone	RCT	Osteoking, Basic therapy	Basic therapy	25mL, qod/12 weeks	FHT, FHR, SRT, VAS
Wang et al. (2023b)	46.20 ± 2.60	47.10 ± 2.80	40/31	39/33	71	72	Tibial	RCT	Osteoking, Basic therapy	Basic therapy	25mL, qod/4 weeks	FHT, FHR
Hao et al. (2022)	67.45 ± 4.99	66.88 ± 5.27	13/20	9/24	33	33	Vertebra	RCT	Osteoking, Basic therapy	Basic therapy	25mL, qod/12 weeks	FHT, FHR, ALP, BGP
He et al. (2022)	68.3 ± 9.2	67.4 ± 8.9	28/10	26/9	38	35	Hip	RCT	Osteoking	Taohong siwu decoction	25mL, qod/4 weeks	FHT, SRT, ERT
Li et al. (2021)	42.41 ± 8.32	40.22 ± 10.85	39/33	42/30	72	72	Tibial	RCT	Osteoking, Basic therapy	Basic therapy	25mL, qod/4 weeks	FHT, FHR, SRT, BGP, ALP
Han and Liu. (2021)	50.84 ± 5.06	50.29 ± 4.94	23/9	24/7	32	31	Hip	RCT	Osteoking	Jiegu Qili tablet	25mL, qod/4 weeks	VAS, SRT
He et al. (2020)	44.3 ± 9.44	44.1 ± 10.27	24/8	23/10	32	33	Tibial	RCT	Osteoking	Taohong siwu decoction	25mL, qod/4 weeks	FHT, FHR, SRT, ERT, VAS, BGP, ALP
Shen (2015)	15∽88	13∽87	35/11	36/10	46	46	Rib	RCT	Osteoking, Basic therapy	Basic therapy	25mL, qod/3 weeks	FHT, FHR, VAS, ALP
Zhu (2014)	39.75 ± 9.57	39.92 ± 9.97	6/6	7/5	12	12	Femur	RCT	Osteoking, Basic therapy	Basic therapy	25mL, qod/8 weeks	FHT, FHR
Zhang et al. (2013)	18∽72	15∽69	39/24	41/22	63	63	Radius	RCT	Osteoking, Basic therapy	Basic therapy	25mL, qod/5 weeks	FHT, FHR, ALP
Luo et al. (2011)	46∽79		NR	NR	42	40	Vertebra	RCT	Osteoking, Basic therapy	Basic therapy	25mL, qod/2 weeks	VAS, BMD
Hu et al. (2005)	15∽85	10∽77	39/33	29/25	72	54	Tibial	RCT	Osteoking	Sanqi Tablets	25mL, qod/3 weeks	FHT, FHR, VAS

### 3.3 Risk of bias

**FIGURE 2:**
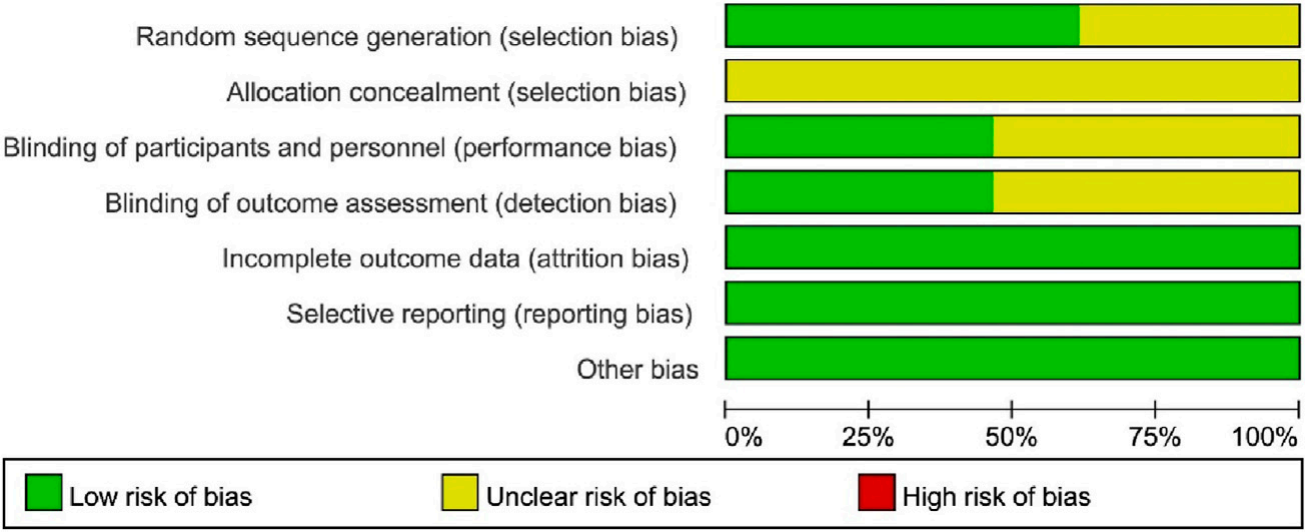
Risk of bias graph.

**FIGURE 3:**
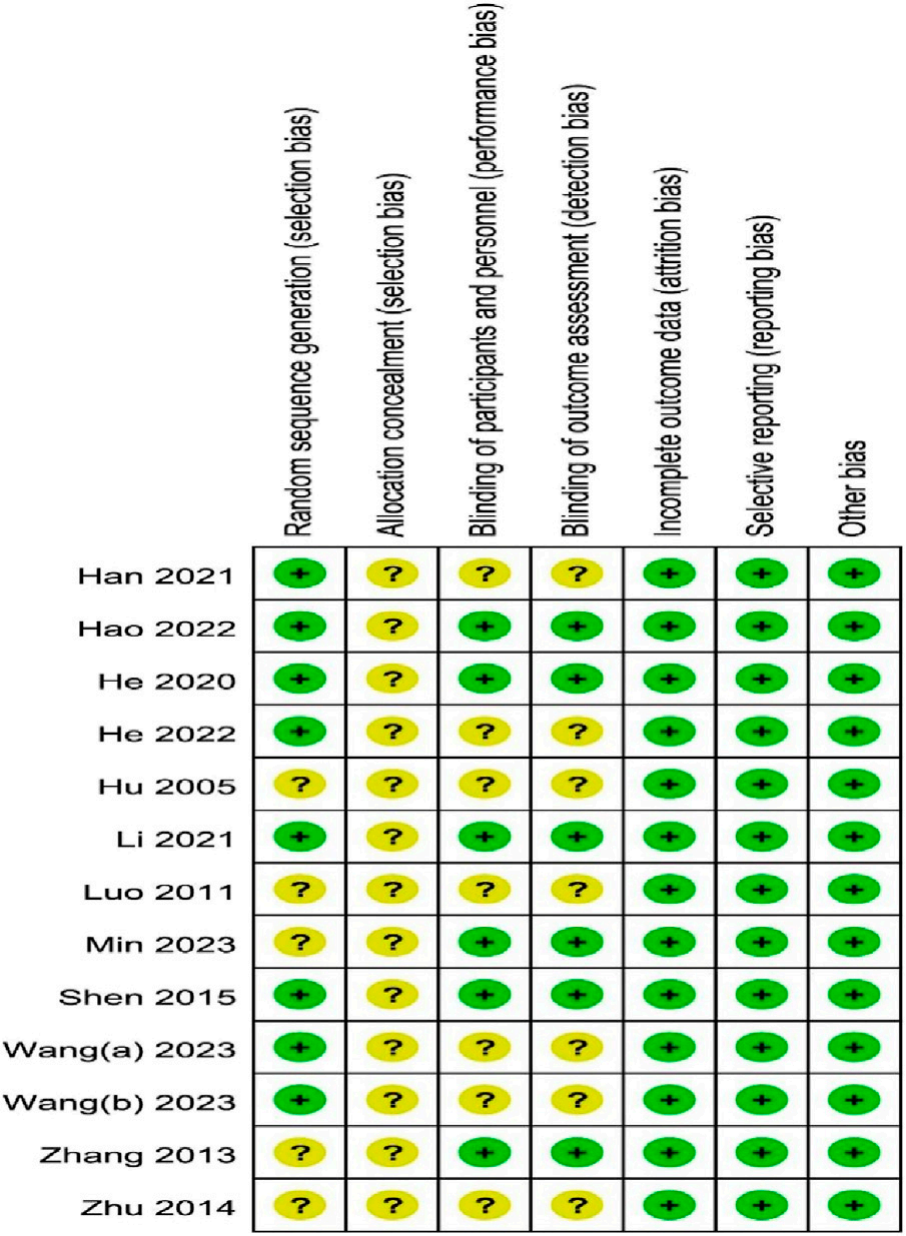
Risk of bias summary.

Seven studies (He et al., 2020; Han and Liu, 2021; Li et al., 2021; Hao et al., 2022; He et al., 2022; Wang et al., 2023a; Wang et al., 2023b) were conducted using a random number table and one trials (Shen, 2015) adopted the lottery method. The remaining studies did not mention the specific randomization methods and were considered to have an unclear risk of bias. Most studies lack a full description of allocation concealment; therefore, the project is mainly assessed as having an “unclear risk of bias.” Although blinding was not reported in some studies, it is unlikely that the assessment of outcome indicators would be compromised by unblinding because some outcome indicators require laboratory instrumentation. Therefore, blinding of participants and personnel, as well as blinding of outcome assessments, was categorized as “low risk of bias.” All included studies reported data for each of the primary outcome indicators in a complete manner, including the number of lost visits and dropouts and the reasons for them; therefore, their attrition bias was assessed as having a low risk of bias. Among the 13 RCTs, we did not find any investigators funded by Osteoking Pharmaceuticals, nor did we find any other potential risks of bias. Therefore, we rated them as “low risk.” The risk of bias was shown in Figure 2 and Figure 3.

### 3.4 Primary outcomes

#### 3.4.1 Fracture healing time

**FIGURE 4:**
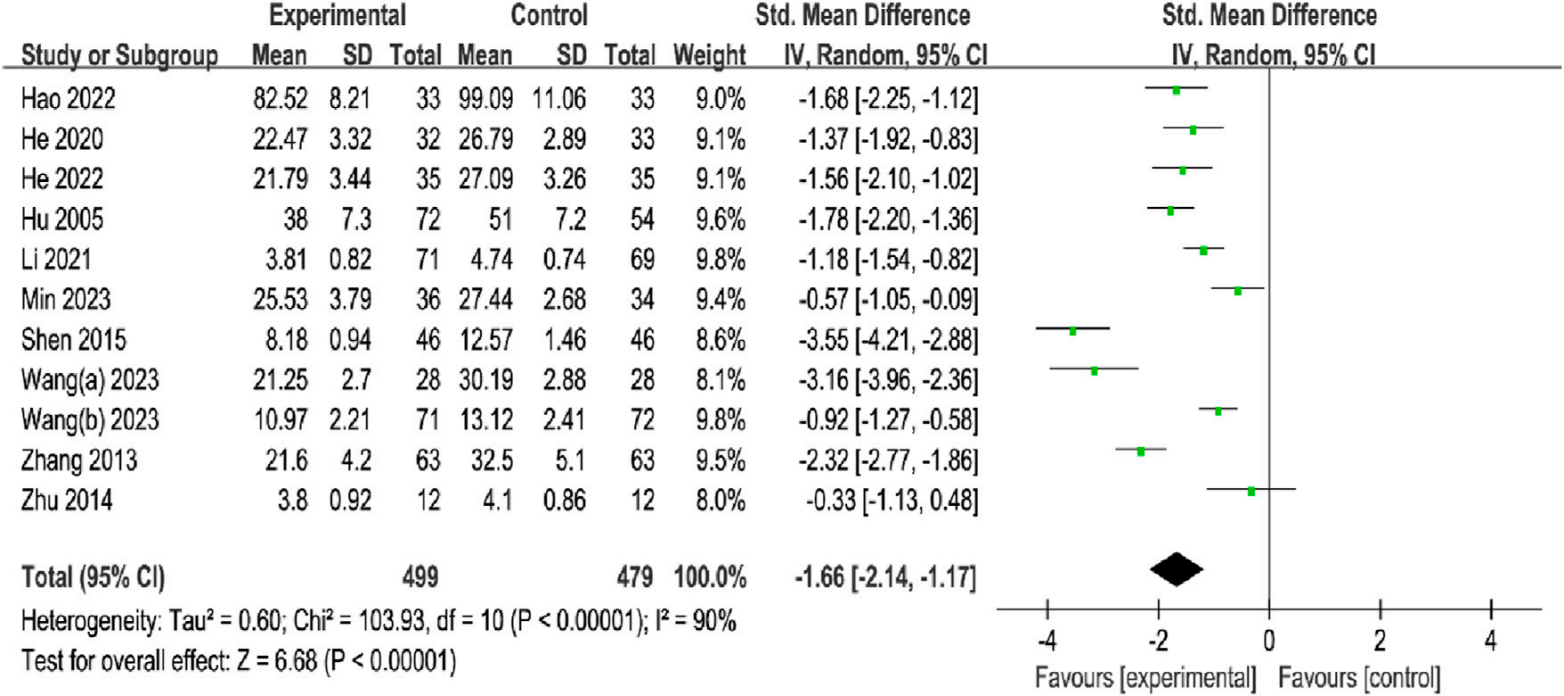
Meta-analysis and forest plot for fracture healing time.

A total of eleven studies, with 499 participants in the OK group and 479 participants in the CG, reported the FHT. The meta-analysis showed that the OK group may be more favorable for shortening the FHT than the CG using the random-effects model (SMD = −1.66%, 95% CI [−2.14, −1.17], *p* < 0.00001; Figure 4). Subgroup analyses by fracture site showed that the OK group may have a significant advantage over CG in reducing FHT at different sites (Limbs long backbone: SMD = −1.57, 95% CI [−2.10, −1.03], *p* < 0.00001; Vertebrae: SMD = −1.68, 95% CI [−2.25, −1.12], *p* < 0.00001; Hip: SMD = −1.06, 95% CI [−2.03, −0.09], *p* = 0.03; Rib: SMD = −3.55, 95% CI [−4.21, −2.88], *p* < 0.00001; Table 2; Supplementary Figure S1). In addition, subgroup analyses based on intervention modality suggested that OK in combination with conventional treatment may be superior to conventional treatment, and OK group may be more effective than other TCM therapies (OK + Basic therapy vs. Basic therapy: SMD = −1.86, 95% CI [−2.60, −1.13], *p* < 0.00001; OK vs. Other Chinese Medicine Therapy (OCMT): SMD = −1.32, 95% CI [−2.14, −1.17], *p* < 0.00001; Table 2; Supplementary Figure S2). In the subgroup analysis of treatment time, the result showed that the OK group may have a substantial benefit over CG, regardless of the duration of treatment (≤4 weeks: SMD = −1.53%, 95% CI [−2.09, −0.97], *p* < 0.00001; >4 weeks: SMD = −1.88%, 95% CI [−2.84, −0.92], *p* < 0.00001; Table 2; Supplementary Figure S3).

**TABLE 2:** Subgroup analysis results.

Outcome	Subgroup	Type	Trials	Participants	Overall effect	Effects mode	*I* ^*2*^	*P*	Figure (S)
FHT	Fracture site	Limbs long backbone	7	680	SMD: −1.57 [-2.10, −1.03]	Random	89	<0.00001	Supplementary Figure S1
		Vertebrae	1	66	SMD: −1.68 [−2.25, −1.12]	Random	-	<0.00001	
		Hip	2	140	SMD: −1.06 [−2.03, −0.09]	Random	86	= 0.03	
		Rib	1	92	SMD: −3.35 [−4.21, −2.88]	Random	-	<0.00001	
	Intervention	OK + BT vs. BT	7	647	SMD: −1.86 [−2.60, −1.13]	Random	93	<0.00001	Supplementary Figure S2
		OK vs. OCMT	4	331	SMD: −1.32 [−1.87, −0.78]	Random	80	<0.00001	
	Treatment duration	≤4 weeks	7	706	SMD: −1.53 [−2.09, −0.97]	Random	91	<0.00001	Supplementary Figure S3
		>4 weeks	4	272	SMD: −1.88 [−2.84, −0.92]	Random	89	= 0.0001	
FHR	Fracture site	Limbs long backbone	7	680	OR:4.18 [2.53, 6.91]	Fixed	0	<0.00001	Supplementary Figure S4
		Vertebrae	1	66	OR: 10.22 [0.53, 197.89]	Fixed	-	0.12	
		Rib	1	24	OR:3.26 [0.12, 88.35]	Fixed	-	0.48	
	Intervention	OK + BT vs. BT	7	579	OR:3.57 [2.06,6.20]	Fixed	0	<0.00001	Supplementary Figure S5
		OK vs. OCMT	2	191	OR:8.17 [2.71,24.66]	Fixed	0	0.002	
	Treatment duration	≤4 weeks	5	498	OR:5.12 [2.67, 9.82]	Fixed	0	<0.00001	Supplementary Figure S6
		>4 weeks	4	272	OR:3.35 [1.59, 7.07]	Fixed	0	= 0.002	
SRT	Fracture site	Limbs long backbone	3	261	SMD: −1.32 [−1.77, −0.87]	Random	60	<0.00001	Supplementary Figure S7
		Hip	3	206	SMD: −1.20 [−1.50, −0.90]	Random	0	<0.00001	
	Intervention	OK + BT vs. BT	2	196	SMD: −1.44 [−2.24, −0.64]	Random	79	0.0004	Supplementary Figure S8
		OK vs. OCMT	4	271	SMD: −1.19 [-1.45, −0.93]	Random	0	<0.00001	
	Treatment duration	≤4 weeks	5	411	SMD: −1.15 [−1.36, −0.94]	Random	0	<0.00001	Supplementary Figure S9
		>4 weeks	1	56	SMD: −1.89 [−2.53, −1.25]	Random	-	<0.00001	
VAS	Fracture site	Limbs long backbone	2	188	SMD: −2.40 [−3.57, −1.22]	Random	96	<0.0001	Supplementary Figure S10
		Hip	1	63	SMD: −0.57 [−0.94, −0.20]	Random	-	= 0.002	
		Vertebrae	1	82	SMD: −1.00 [−1.47, −0.53]	Random	-	<0.0001	
		Rib	1	92	SMD: −0.07 [−0.54, 0.40]	Random	-	= 0.77	
	Intervention	OK + BT vs. BT	3	230	SMD: −0.98 [−2.06, 0.10]	Random	96	= 0.07	Supplementary Figure S11
		OK vs. OCMT	2	195	SMD: −1.79 [−4.19, 0.61]	Random	98	= 0.14	
	Treatment duration	≤4 weeks	4	369	SMD: −1.16 [−2.38, −0.06]	Random	97	= 0.06	Supplementary Figure S12
		>4 weeks	1	56	SMD: −1.82 [−1.97, −1.67]	Random	-	<0.00001	
BGP	Fracture site	Limbs long backbone	2	205	SMD: 0.63 [0.39, 0.86]	Fixed	0	<0.00001	Supplementary Figure S13
		Hip	1	70	SMD: 0.77 [0.61, 0.93]	Fixed	-	<0.00001	
		Vertebrae	1	66	SMD: 0.70 [0.48, 0.92]	Fixed	0	<0.00001	
	Intervention	OK + BT vs. BT	2	206	SMD: 0.71 [0.49, 0.93]	Fixed	0	<0.00001	Supplementary Figure S14
		OK vs. OCMT	2	135	SMD: 0.72 [0.59, 0.85]	Fixed	17	<0.00001	
	Treatment duration	≤4 weeks	3	275	SMD: 0.73 [0.60, 0.86]	Fixed	0	<0.00001	Supplementary Figure S15
		>4 weeks	1	66	SMD: 0.70 [0.48, 0.92]	Fixed	-	<0.00001	
ALP	Fracture site	Limbs long backbone	3	331	SMD:16.54 [10.54,22.55]	Random	88	<0.00001	Supplementary Figure S16
		Hip	1	70	SMD: 13.06 [9.12, 17.00]	Random	-	<0.00001	
		Rib	1	92	SMD:40.19 [37.19,43.19]	Random	-	<0.00001	
		Vertebrae	1	66	SMD: 6.49 [3.25, 9.73]	Random	-	<0.00001	
	Intervention	OK + BT vs. BT	4	349	SMD: 21.30 [4.94, 37.66]	Random	99	= 0.01	Supplementary Figure S17
		OK vs. OCMT	2	210	SMD:12.19 [10.43,13.94]	Random	0	<0.00001	
	Treatment duration	≤4 weeks	4	367	SMD:20.46 [6.18, 34.75]	Random	99	= 0.005	Supplementary Figure S18
		>4 weeks	2	192	SMD:14.10 [-0.99, 29.18]	Random	97	= 0.07	

#### 3.4.2 Fracture healing rate

**FIGURE 5:**
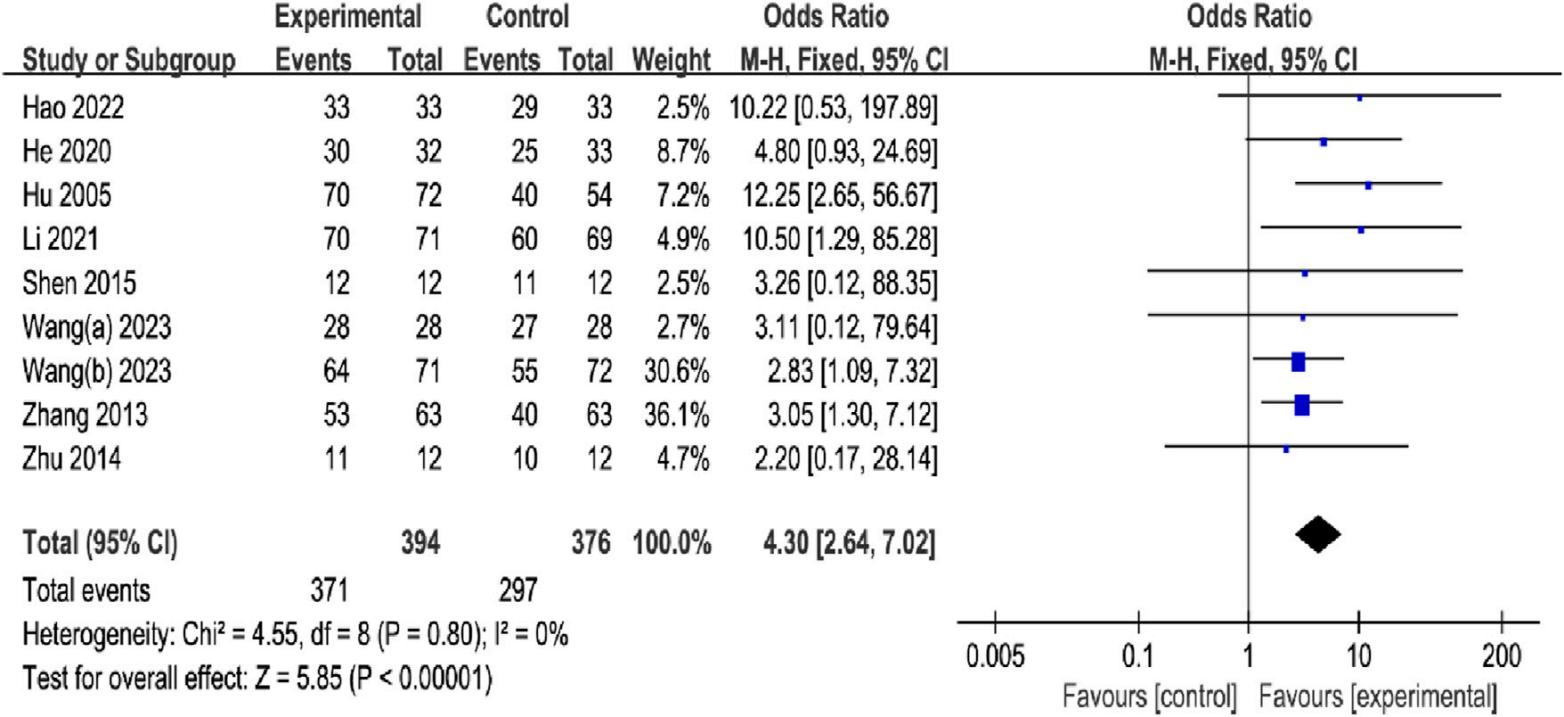
Meta-analysis and forest plot for fracture healing rate.

A total of nine studies, with 394 participants in the OK groups and 376 participants in the CG, reported the FHR. The meta-analysis showed that OK group was more favorable for increasing fracture healing rate compared to CG with the fixed effects model (OR = 4.30%, 95% CI [2.64, 7.02], *p* < 0.00001; Figure 5). Subgroup analyses by fracture site showed that the OK group may have a significant advantage over CG in improving the fracture healing rate at Limbs long backbone (OR = −1.57%, 95% CI [−2.10, −1.03], *p* < 0.00001**;** Table 2; Supplementary Figure S4). However, the OK group showed no obvious improvement in healing rates of the vertebrae and rib (Table 2; Supplementary Figure S4). In addition, subgroup analyses based on intervention modality suggested that OK in combination with conventional treatment may be superior to conventional treatment, and OK group was more effective than other Chinese medicine therapies (OK + Basic therapy vs. Basic therapy: OR = 3.57%, 95% CI [ 2.06,6.20], *p* < 0.00001; OK vs. OCMT: OR = 8.17%, 95% CI [2.71,24.66], *p* = 0.0002; Table 2; Supplementary Figure S5). In the subgroup analysis of treatment time, the result showed that the OK group may have a substantial benefit over CG, regardless of the duration of treatment (≤4 weeks: OR = 5.12%, 95% CI [2.67,9.82], *p* < 0.00001; >4 weeks: OR = 3.35%, 95% CI [1.59, 7.07], *p* < 0.00001; Table 2; Supplementary Figure S6).

### 3.5 Secondary outcomes

#### 3.5.1 Swelling regression time

**FIGURE 6:**
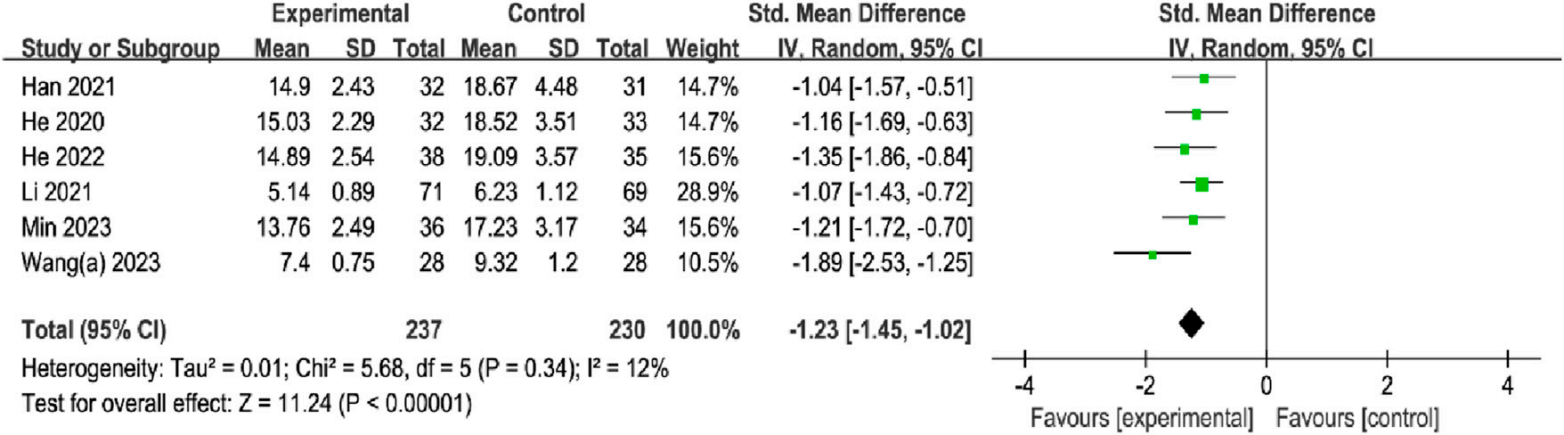
Meta-analysis and forest plot for swelling regression time.

A total of six studies, with 237 participants in the OK group and 230 participants in the CG, reported the SRT. The meta-analysis showed that the OK group was more favorable for the reduction of swelling regression time compared to the CG with the fixed effects model (SMD = −1.23%, 95% CI [−1.45, −1.02], *p* < 0.00001; Figure 6). In subgroup analyses, there were significant differences between fracture site, intervention method, and treatment duration subgroups (Table 2; Supplementary Figures S7–9).

#### 3.5.2 Ecchymosis regression time

**FIGURE 7:**
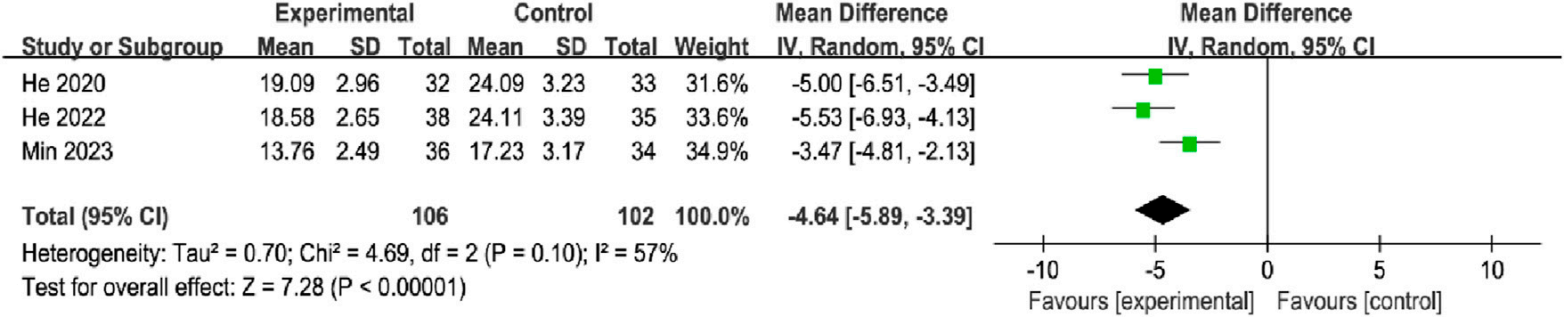
Meta-analysis and forest plot for ecchymosis regression time.

A total of three studies, with 106 participants in the OK groups and 102 participants in the CG, reported the ERT. The meta-analysis showed that the OK group may be more favorable for reducing ecchymosis regression time compared to the CG group with the random effects model (MD = −4.64, 95% CI [−5.89, −3.39], *p* < 0.00001; Figure 7).

#### 3.5.3 Visual analogue scale

**FIGURE 8:**
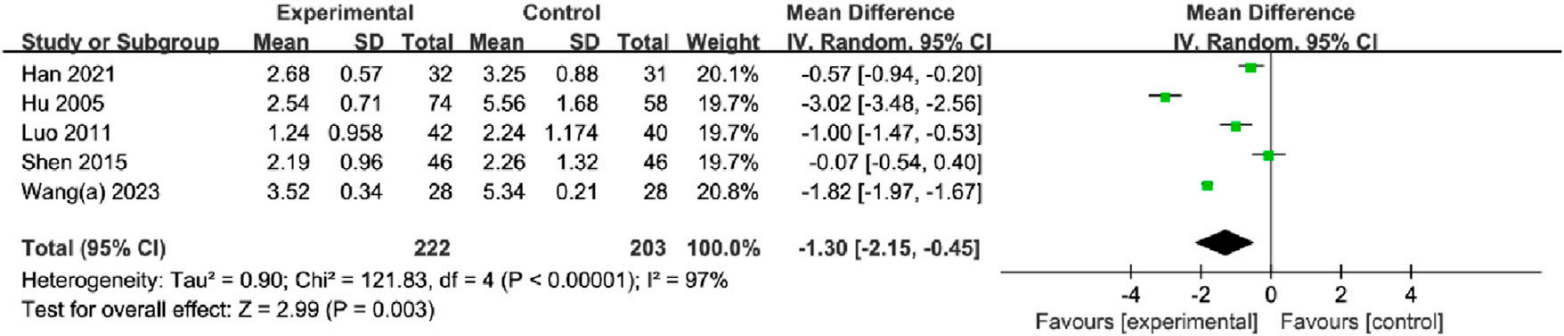
Meta-analysis and forest plot for visual analogue scale.

A total of five studies, with 222 participants in the OK group and 203 participants in the CG, reported the VAS. The meta-analysis showed that the OK group may have a significant reduction in pain compared to the CG group with the random effects model (MD = −1.30%, 95% CI [−2.15, −0.45], *p* = 0.003; Figure 8). Subgroup analyses by fracture site showed that the OK group may have a significant advantage over CG in relieving pain at Limbs long backbone, Hip, and vertebrae, however, there was no obvious difference in the ribs (Table 2; Supplementary Figure S10). Additionally, there was no significant difference between the two groups in the subgroup analysis based on the intervention method (Table 2; Supplementary Figure S11). According to the subgroup analysis based on treatment duration, there was no significant difference in short-term pain relief efficacy between the OK group and CG (Table 2; Supplementary Figure S12).

#### 3.5.4 Bone Gla-protein

**FIGURE 9:**
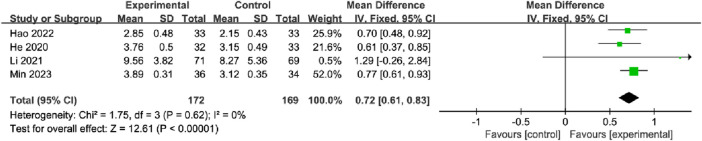
Meta-analysis and forest plot for Bone gla-protein.

A total of four studies, with 172 participants in the OK group and 169 participants in the CG, reported the BGP. The meta-analysis showed that OK treatment may have a significant increase in BGP vs. CG with the fixed effects model (MD = 0.72%, 95% CI [0.61, 0.83], *p* < 0.00001; Figure 9). In the subgroup analyses, significant differences were observed between the fracture site, intervention method, and treatment duration subgroups (Table 2; Supplementary Figures S13–15).

#### 3.5.5 Alkaline phosphatase

**FIGURE 10:**
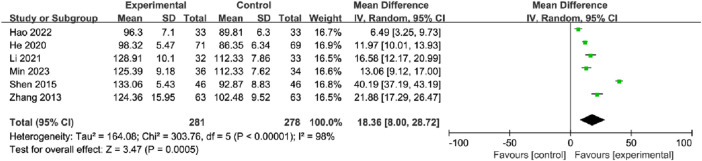
Meta-analysis and forest plot for alkaline phosphatase.

A total of six studies, with 281 participants in the OK group and 278 participants in the CG, reported the ALP. The meta-analysis showed that OK treatment may have a significant improvement in ALP compared with that in the CG using the random-effects model (MD = 18.36, 95% CI [8.00, 28.72], *p* = 0.0005; Figure 10). In subgroup analyses, there were significant differences between fracture site, intervention method, and treatment duration subgroups (Table 2; Supplementary Figures S16–18).

#### 3.5.6 Type I procollagen carboxy-terminal peptide

**FIGURE 11:**
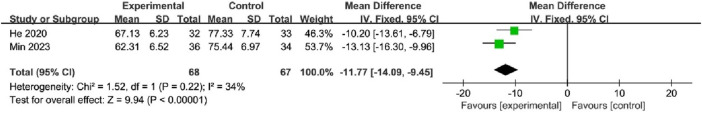
Meta-analysis and forest plot for type I procollagen carboxy-terminal peptide.

A total of two studies, with 68 participants in the OK group and 67 participants in the CG, reported PICP. The meta-analysis showed that OK treatment may have a significant reduction in PICP compared with that in the CG with the fixed-effects model (MD = −11.77%, 95% CI [−14.09, −9.45], *p* < 0.00001; Figure 11).

#### 3.5.7 Adverse events

One trial (Wang et al., 2023a) reported two cases of dry mouth, one case of oral ulcer, two cases of skin itching, and four cases of gastrointestinal reactions in the OK group, and two cases of dry mouth, two cases of oral ulcer, and three cases of gastrointestinal reactions in the CG. There was no significant difference in the incidence of adverse reactions between the two groups. In one study (Shen, 2015), two patients in the OK group reported adverse effects, such as dry mouth, that were not treated and resolved spontaneously after 2–6 h. None of the ten RCTs reported adverse event information for all included studies (Luo et al., 2011; Zhang et al., 2013; Zhu, 2014; He et al., 2020; Han and Liu, 2021; Li et al., 2021; Hao et al., 2022; He et al., 2022; Liu et al., 2022; Wang et al., 2023b; Min, 2023). In addition, in another study (Hu et al., 2005), five patients experienced side effects, such as dizziness, but the symptoms were mild and resolved on their own.

### 3.6 Sensitivity analysis

In this review, we performed sensitivity analyses of the primary outcomes by removing trials individually. The results showed that the pooled analysis results were stable for the primary outcomes (Supplementary Table S1).

### 3.7 Publication bias

**FIGURE 12:**
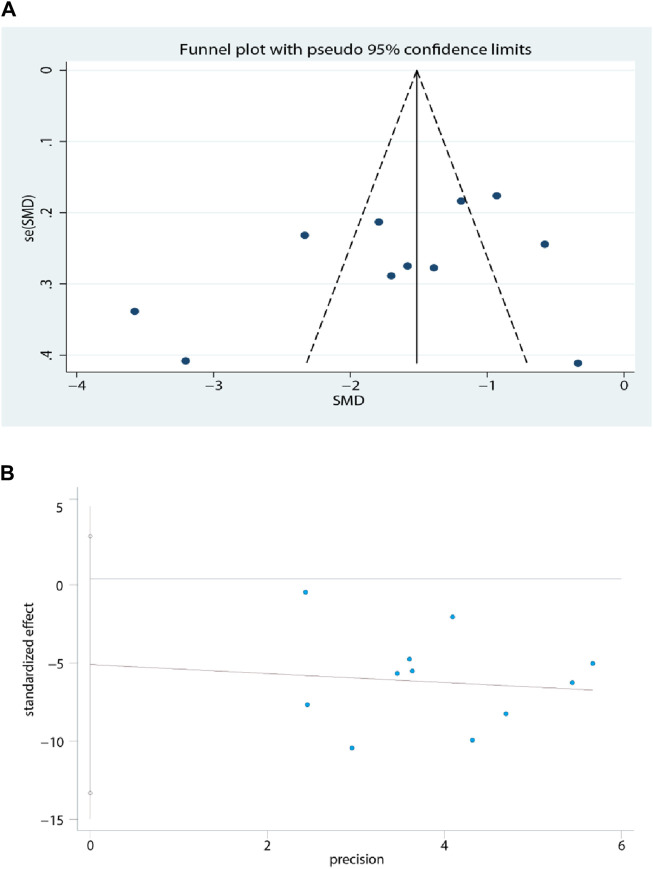
**(A)** The funnel plot for the FHT. **(B)** The Eggers’ test for the FHT.

Funnel plots and Egger’s tests were performed only for outcome measures in more than ten studies. The funnel plot for the FHT was symmetric, as shown in Figure 12A. Egger’s test for the FHT indicated no significant publication bias (t = −1.41, *p* = 0.192) in the included studies (Figure 12B).

### 3.8 Evidence quality assessment

A summary of the GRADE results is provided in Table 3. There was a low level of evidence for FHT, FHR, and SRT, and very low evidence for the remaining outcomes based on the GRADE approach.

**TABLE 3:** Quality of evidence.

Outcomes	No of studies (Participants)	Design	Quality assessment					Effect (95% CI)	Certainty
			Risk of bias	Inconsistency	Indirectness	Imprecision	Other considerations (Publication bias)		
FHT	11 studies (978)	RCT	serious	serious	not serious	not serious	suspected	SMD = −1.66, [−2.14, −1.17]	Low
FHR	9 studies (770)	RCT	serious	not serious	not serious	not serious	strongly suspected	OR = 4.30, [2.64, 7.02]	Low
SRT	6 studies (467)	RCT	serious	not serious	not serious	not serious	strongly suspected	SMD = −1.22, [−1.42, −1.02]	Low
ERT	3 studies (208)	RCT	serious	serious	not serious	serious	strongly suspected	MD = −4.64, [−5.89, −3.39]	Very Low
VAS	5 studies (425)	RCT	serious	serious	not serious	not serious	strongly suspected	MD = −1.30, [−2.15, −0.45]	Very Low
BGP	4 studies (341)	RCT	serious	not serious	not serious	serious	strongly suspected	MD = 0.72, [0.61, 0.83]	Very Low
ALP	6 studies (559)	RCT	serious	serious	not serious	not serious	strongly suspected	MD = 18.36, [8.00, 28.72]	Very Low
PICP	2 studies (135)	RCT	serious	not serious	not serious	serious	strongly suspected	MD = −11.77, [−14.09, −9.45]	Very Low

## 4 Discussion

This systematic review and meta-analysis assessed the efficacy and safety of OK for fracture healing. In this review, OK therapy was found to be beneficial and safe for accelerating fracture healing, reducing swelling, eliminating bruising, improving bone metabolism, and promoting postoperative recovery. Fracture healing is influenced by many factors such as fracture site, severity of trauma, chronic illness, malnutrition, and use of certain medications (Rodham et al., 2023). Therefore, although we performed a subgroup analysis of the outcome indicators according to fracture site, intervention type, and treatment course, the heterogeneity of some outcome indicators remained high. It is worth noting that the results of the VAS were not fully consistent with the results of the subgroup analysis based on the treatment course and intervention type.

In the systematic review, we found that the most commonly used dose of OK to promote fracture healing is 25 mL every other day, according to the included literature. Furthermore, the majority of OK courses were less than or equal to 4 weeks. The duration of intervention in the four studies was greater than 4 weeks; two of the studies (Zhu, 2014; Wang et al., 2023b) had a diagnosis of nonunion, one study (Zhang et al., 2013) had a diagnosis of comminuted fractures; and one study (Hao et al., 2022) had a diagnosis of osteoporotic fractures in older patients. The results have shown that a course of OK lasting less than or equal to 4 weeks can effectively promote fracture healing, but nonunion fractures, older patients, or more serious injuries may require a longer period of time. In addition, there were few reports on side effects in the literature included in this review, with only three studies mentioning side effects. No serious side effects were observed in the included studies, and mild side effects that did occur resolved on their own without treatment. However, further research is required to determine the specific causes of the adverse effects of surgical procedures and other medications.

OK has been used for fracture healing in China for more than 600 years. However, no published studies have evaluated the efficacy and safety of OK in fracture healing. According to the theory of Chinese medicine, the healing of bone fractures requires the activation of blood circulation and elimination of blood stasis, as well as the strengthening of tendons and bones. The combination of herbs in OK works to reduce pain in the acute phase and promotes recovery in the remission phase. OK is composed of *C. tinctorius* L., *P. notoginseng* F.H.Chen*, E. ulmoides* Oliv, *P. ginseng* C.A.Mey, *C. reticulata Blanco* D.C., *A. hamosus* L., *D. metel* L., *T. sinensis* W., *S. integrifolium* Oliv., which has the effects of activating blood and replenishing qi, bone and tendon (http://www.worldfloraonline.org, date of visit: 17 November 2023). *Carthamus tinctorius* L. has anti-inflammatory and analgesic effects, and its mechanism is related to increasing the release of interleukin-4 and reducing interleukin-1β (Yousefi et al., 2021). In addition, Hydroxysafflor Yellow A promotes bone mineralization and inhibits bone resorption, thereby reversing glucocorticoid-induced osteoporosis (Nie et al., 2019). The anti-inflammatory effect of *P. notoginseng* F.H.Chen is mainly through inhibiting the secretion of TNF-α and IL-6 in macrophages induced by lipopolysaccharide (Wang et al., 2014). *Citrus reticulata Blanco* D.C. active ingredient tangeretin activates AMPK-PGC1-α pathway and enhances mitochondrial biosynthesis, showing the potential to protect muscles and bones, thereby improving exercise performance (Kou et al., 2018). The chemical constituents of Panax ginseng C.A.Mey regulate and maintain the normal physiological functions of the immune system and promote specific or non-specific immunity (Ratan et al., 2021b). Eucommia ulmoides leaf extract can regulate the diversity of intestinal microflora and increase the content of short-chain fatty acids to exert bone protection (Zhao et al., 2020). *Astragalus hamosus* L. can promote angiogenesis by inhibiting inflammatory response and upregulating the expression of VEGF and p-AKT/AKT proteins (Wang et al., 2021). *Datura metel* L. has the effect of inhibiting cholinergic nerves, which relieves muscle spasms and pain (Ratan et al., 2021b). *Schizophragma integrifolium* Oliv. is mainly composed of terpenoids, aromatic hydroxyls, and other components that have anti-inflammatory, analgesic, and detumescent effects (Zeng et al., 2009). The bioactive substances extracted from *T. sinensis* W. have the effects of anti-tumor, anti-inflammatory and improving immune function (Sun et al., 2021). Pharmacological studies have shown that OK stimulates the Wnt signaling pathway by downregulating the serum levels of Dickkopf-related protein 1 in fractured rabbits to promote bone formation, increase bone mineral density, and treat fractures (Wang et al., 2014). Additionally, it has also been shown that OK can increase levels of basic fibroblast growth factor, platelet-derived growth factor, and vascular endothelial growth factor in fracture rabbits, promoting bone repair and remodeling (Wu and Zhan, 2017). Moreover, the clinical treatment results showed that OK was more effective in improving microcirculation, removing blood clots and metabolites at the fracture site, accelerating soft tissue injury repair and edema absorption, and reducing inflammatory exudate stimulation of nerve endings (Yuan et al., 2019; Cai et al., 2021).

Some limitations of this study are listed below. First, all studies were conducted in China, and research from other countries, regions, and data from other populations were lacking, limiting their applicability. Second, this review selected all fracture types to include all RCTs on OK interventions for fracture healing, which increased the heterogeneity of the results. Third, some studies had small sample sizes and low quality, which negatively impacted the strength of the evidence for the research findings. Therefore, additional original studies with higher quality, standardization, and more fracture types are required to confirm these conclusions.

To improve future trials on this topic, we have some insights. Firstly, to ensure the transparency of clinical trial research and the ethical and scientific nature of the research, the program should be registered on the international clinical trial registration platform. Tests should be reported in detail in accordance with the Consolidated Standard for Reported Trials statement. Secondly, it is recommended that future clinical trials employ the most appropriate randomization method and blind method, and that the sample size be estimated in a reasonable manner. Thirdly, longer follow-up is necessary to determine the potential benefits of OK on the reduction of re-fractures. Fourthly, Patients enrolled in clinical trials should be excluded if they have underlying conditions that interfere with fracture healing. Fifthly, the selection of primary outcome indicators should align with internationally or domestically recognized efficacy evaluation indicators.

## 5 Conclusion

In this review, we found that OK therapy was beneficial and safe for accelerating fracture healing, reducing swelling, eliminating bruising, and improving bone metabolism. However, the meta-analysis results do not support OK treatment for improving the FHR at all fracture sites and reducing pain across all fracture sites. Current evidence suggests that OK may be an effective treatment option for patients with fractures. Further well-designed, high-quality studies are needed to validate these findings.

## Data Availability

The original contributions presented in the study are included in the article/Supplementary Material, further inquiries can be directed to the corresponding authors.
