# Supplementary material for: Efficacy and safety of Osteoking on fracture healing: a systematic review and meta-analysis
Source: Front Pharmacol. 2024 Jun 10;15:1363421. doi: 10.3389/fphar.2024.1363421 (PMC11194365; doi:10.3389/fphar.2024.1363421)
Supplement: Supplementary file 1 [file DataSheet1.pdf]

Supplementary Material S1. Details of the Osteoking.

Name (English/Chinese)	Source	SFDA approval number	Compositions	Description	Extraction procedure	Actions	Adverse reaction	Dosage and administration	Quality control reported? (Y/N)	Chemical analysis reported?
Osteoking/Henggu Gushangyu Heji	Sailing Pharmaceutical Technology Group Co.，Ltd	Z20025103	Carthamus tinctorius L.<br>Panax notoginseng F.H.Chen<br>Eucommia ulmoides Oliv<br>Panax ginseng C.A.Mey<br>Citrus reticulata Blanco D.C.<br>Trionyx sinensis W.<br>Astragalus hamosus L.<br>Datura metel L., Trionyx sinensis W.<br>Schizophragma integrifolium Oliv.	This product is in oral dosage form; Odor : spicy; taste: slightly bitter.	Add water to soak for 12 hours, decocted three times, each time for 1 hour, and collect the distilled liquid for cold storage. The decoction was combined and filtered. The filtrate was concentrated to a relative density of 1.03~1.04 (50°C), centrifuged, allowed to stand for 12 hours, and filtered. The filtrate was mixed with the above distilled liquid, added with ethyl p-hydroxybenzoate 0.4g, adjusted to pH 4.0 ~ 6.0 with 0.05 % sodium bicarbonate solution, added with water to 1000ml, filtered, and filled to obtain.	Activate blood and replenish qi, bone and tendon, promote fracture healing.	Post-marketing adverse reaction monitoring revealed cases of dry mouth, mild dizziness, nausea, vomiting, diarrhea, gastric upset, abdominal pain, rash, itching, palpitations, and fatigue after taking the drug.	Oral, 25ml once, once every other day, 12 days for a course of treatment.	Y-prepared according to Pharmacopedia of the People's Republic of China (2000) and National Medical Products Administration Standards:WS-10094（ZD-0094)-2002-2012Z.	Y-HPLC [Detail information can be got from Pharmacopoeia of the People's Republic of China (2000) (Part I, Page number 1356).

Supplementary Material S2. The search strategy in this review.

Search strategy for PubMed (47)

Search	Terms
#1	("Fracture Healing"[Mesh]) OR (((((((((((((((Fracture Healings) OR (Healing, Fracture)) OR (Healings, Fracture)) OR (Bone Fracture)) OR (Fracture, Bone)) OR (Broken Bones)) OR (Bone, Broken)) OR (Broken Bone)) OR (Bone Fractures)) OR (Spiral Fractures)) OR (Fracture, Spiral)) OR (Fractures, Spiral)) OR (Spiral Fracture)) OR (Torsion Fractures)) OR (Fracture, Torsion)) OR (Torsion Fracture))
#2	("Drugs, Chinese Herbal"[Mesh]) OR (((((((Osteoking) OR (Chinese Drugs, Plant)) OR (Chinese Herbal Drugs)) OR (Herbal Drugs, Chinese)) OR (Plant Extracts, Chinese)) OR (Chinese Plant Extracts)) OR (Extracts, Chinese Plant))
#3	((randomized controlled trial[Publication Type]) OR (randomized[Title/Abstract])) OR (placebo[Title/Abstract])
#4	#1 AND #2 AND #3

Search strategy for Embase (104)

Search	Terms
#1	'Chinese medicine'/exp
#2	'Chinese Drugs, Plant' OR 'Chinese Herbal Drugs' OR 'Herbal Drugs, Chinese' OR 'Plant Extracts, Chinese' OR 'Chinese Plant Extracts' OR 'Extracts, Chinese Plant' OR 'Osteoking'
#3	#1 OR #2
#4	'fracture healing'/exp
#5	'Fracture Healings' OR 'Healing, Fracture' OR 'Healings, Fracture' OR 'Bone Fracture' OR 'Fracture, Bone' OR 'Broken Bones' OR 'Bone, Broken' OR 'Broken Bone' OR 'Bone Fractures' OR 'Spiral Fractures' OR 'Fracture, Spiral' OR 'Fractures, Spiral' OR 'Spiral Fracture' OR 'Torsion Fractures' OR 'Fracture, Torsion' OR 'Torsion Fracture'
#6	#4 OR #5
#7	'clinical trial'/exp OR 'clinical trial' OR (('clinical' OR 'clinical'/exp OR clinical) AND ('trial' OR 'trial'/exp OR trial)) OR (randomized AND controlled AND ('trial'/exp OR trial)) OR trials
#8	#3 AND #6 AND #7

Search strategy for CENTRAL (27)

Search	Terms

#1	(Chinese Herbal Drugs):ti,ab,kw
#2	(Chinese Drugs, Plant):ti,ab,kw
#3	(Herbal Drugs, Chinese):ti,ab,kw
#4	(Extracts, Chinese Plant):ti,ab,kw
#5	(Chinese Plant Extracts):ti,ab,kw
#6	(Plant Extracts, Chinese):ti,ab,kw
#7	(Osteoking):ti,ab,kw
#8	MeSH descriptor: [Drugs, Chinese Herbal] explode all trees
#9	#1 OR #2 OR #3 OR #4 OR #5 OR #6 OR #7
#10	((Healing, Fracture) OR (Healings, Fracture) OR (Fracture Healings) OR (Bones, Broken) OR (Broken Bone) OR (Broken Bones) OR (Bone Fracture) OR (Bone Fractures) OR (Bone, Broken) OR (Fracture, Bone) OR (Fracture, Torsion) OR (Fracture, Spiral) OR (Fractures, Torsion) OR (Torsion Fractures) OR (Spiral Fractures) OR (Torsion Fracture) OR (Fractures, Spiral) OR (Spiral Fracture)):ti,ab,kw
#11	#9 AND #10

Search strategy for CNKI（52）

(SU%=恒古骨伤愈合剂 + 恒古 + 骨王) AND (SU%=骨折 + 骨愈合 + 骨折痊愈)

Search strategy for WanFang（49）

主题: ("恒古骨伤愈合剂" OR "恒古"OR "骨王") and 主题: ("骨折" OR"骨愈合" OR"骨折痊愈")

Search strategy for VIP（74）

(U=恒古骨伤愈合剂 OR 恒古 OR 骨王) AND (U=骨折 OR 骨愈合 OR 骨折痊愈)

Search strategy for CBM（36）

("骨折"[全部字段:智能] OR "骨愈合"[全部字段:智能] OR "骨折痊愈"[全部字段:智能]) AND ("恒古骨伤愈合剂"[全部字段:智能] OR "恒古"[全部字段:智能] OR "骨王"[全部字段:智能])

Supplementary material S3. Summary of composition characteristics of preparations in all included original studies.

Study	Formulation	Source	Species, concentration	Quality control reported? (Y/N)
Min 2023	Osteoking	Sailing Pharmaceutical Technology Group Co.，Ltd	Carthamus tinctorius L., Panax notoginseng F.H.Chen, Eucommia ulmoides Oliv, Panax ginseng C.A.Mey, Citrus reticulata Blanco D.C., Astragalus hamosus L.，Datura metel L., Trionyx sinensis W. and Schizophragma integrifolium Oliv.	Y-prepared according to Pharmacopedia of the People's Republic of China (2000) and National Medical Products Administration Standards:WS-10094（ZD-0094)-2002-2012Z.
Wang(a) et al. 2023	Osteoking	Sailing Pharmaceutical Technology Group Co.，Ltd	Carthamus tinctorius L., Panax notoginseng F.H.Chen, Eucommia ulmoides Oliv, Panax ginseng C.A.Mey, Citrus reticulata Blanco D.C., Astragalus hamosus L.，Datura metel L., Trionyx sinensis W. and Schizophragma integrifolium Oliv.	Y-prepared according to Pharmacopedia of the People's Republic of China (2000) and National Medical Products Administration Standards:WS-10094（ZD-0094)-2002-2012Z.
Wang(b) et al. 2023	Osteoking	Sailing Pharmaceutical Technology Group Co.，Ltd	Carthamus tinctorius L., Panax notoginseng F.H.Chen, Eucommia ulmoides Oliv, Panax ginseng C.A.Mey, Citrus reticulata Blanco D.C., Astragalus hamosus L.，Datura metel L., Trionyx sinensis W. and Schizophragma integrifolium Oliv.	Y-prepared according to Pharmacopedia of the People's Republic of China (2000) and National Medical Products Administration Standards:WS-10094（ZD-0094)-2002-2012Z.
Hao et al. 2022	Osteoking	Sailing Pharmaceutical Technology Group Co.，Ltd	Carthamus tinctorius L., Panax notoginseng F.H.Chen, Eucommia ulmoides Oliv, Panax ginseng C.A.Mey, Citrus reticulata Blanco D.C., Astragalus hamosus L.，Datura metel L., Trionyx sinensis W. and Schizophragma integrifolium Oliv.	Y-prepared according to Pharmacopedia of the People's Republic of China (2000) and National Medical Products Administration Standards:WS-10094（ZD-0094)-2002-2012Z.
He et al. 2022	Osteoking	Sailing Pharmaceutical Technology Group Co.，Ltd	Carthamus tinctorius L., Panax notoginseng F.H.Chen, Eucommia ulmoides Oliv, Panax ginseng	Y-prepared according to Pharmacopedia of the People's Republic of China (2000) and

			C.A.Mey, Citrus reticulata Blanco D.C., Astragalus hamosus L.，Datura metel L., Trionyx sinensis W. and Schizophragma integrifolium Oliv.	National Medical Products Administration Standards:WS-10094（ZD-0094)-2002-2012Z.
Li et al. 2021	Osteoking	Sailing Pharmaceutical Technology Group Co.，Ltd	Carthamus tinctorius L., Panax notoginseng F.H.Chen, Eucommia ulmoides Oliv, Panax ginseng C.A.Mey, Citrus reticulata Blanco D.C., Astragalus hamosus L.，Datura metel L., Trionyx sinensis W. and Schizophragma integrifolium Oliv.	Y-prepared according to Pharmacopedia of the People's Republic of China (2000) and National Medical Products Administration Standards:WS-10094（ZD-0094)-2002-2012Z.
Han et al. 2021	Osteoking	Sailing Pharmaceutical Technology Group Co.，Ltd	Carthamus tinctorius L., Panax notoginseng F.H.Chen, Eucommia ulmoides Oliv, Panax ginseng C.A.Mey, Citrus reticulata Blanco D.C., Astragalus hamosus L.，Datura metel L., Trionyx sinensis W. and Schizophragma integrifolium Oliv.	Y-prepared according to Pharmacopedia of the People's Republic of China (2000) and National Medical Products Administration Standards:WS-10094（ZD-0094)-2002-2012Z.
He et al. 2020	Osteoking	Sailing Pharmaceutical Technology Group Co.，Ltd	Carthamus tinctorius L., Panax notoginseng F.H.Chen, Eucommia ulmoides Oliv, Panax ginseng C.A.Mey, Citrus reticulata Blanco D.C., Astragalus hamosus L.，Datura metel L., Trionyx sinensis W. and Schizophragma integrifolium Oliv.	Y-prepared according to Pharmacopedia of the People's Republic of China (2000) and National Medical Products Administration Standards:WS-10094（ZD-0094)-2002-2012Z.
Shen 2015	Osteoking	Sailing Pharmaceutical Technology Group Co.，Ltd	Carthamus tinctorius L., Panax notoginseng F.H.Chen, Eucommia ulmoides Oliv, Panax ginseng C.A.Mey, Citrus reticulata Blanco D.C., Astragalus hamosus L.，Datura metel L., Trionyx sinensis W. and Schizophragma integrifolium Oliv.	Y-prepared according to Pharmacopedia of the People's Republic of China (2000) and National Medical Products Administration Standards:WS-10094（ZD-0094)-2002-2012Z.
Zhu 2014	Osteoking	Sailing Pharmaceutical Technology Group Co.，Ltd	Carthamus tinctorius L., Panax notoginseng F.H.Chen, Eucommia ulmoides Oliv, Panax ginseng C.A.Mey, Citrus reticulata Blanco D.C., Astragalus hamosus L.，Datura metel L., Trionyx sinensis W. and	Y-prepared according to Pharmacopedia of the People's Republic of China (2000) and National Medical Products Administration Standards:WS-10094

			Schizophragma integrifolium Oliv.	(ZD-0094)-2002-2012Z.
Zhang et al. 2013	Osteoking	Sailing Pharmaceutical Technology Group Co.，Ltd	Carthamus tinctorius L., Panax notoginseng F.H.Chen, Eucommia ulmoides Oliv, Panax ginseng C.A.Mey, Citrus reticulata Blanco D.C., Astragalus hamosus L.，Datura metel L., Trionyx sinensis W. and Schizophragma integrifolium Oliv.	
Luo et al. 2011	Osteoking	Sailing Pharmaceutical Technology Group Co.，Ltd	Carthamus tinctorius L., Panax notoginseng F.H.Chen, Eucommia ulmoides Oliv, Panax ginseng C.A.Mey, Citrus reticulata Blanco D.C., Astragalus hamosus L.，Datura metel L., Trionyx sinensis W. and Schizophragma integrifolium Oliv.	Y-prepared according to Pharmacopedia of the People's Republic of China (2000) and National Medical Products Administration Standards:WS-10094 (ZD-0094)-2002-2012Z.
Hu et al. 2005	Osteoking	Sailing Pharmaceutical Technology Group Co.，Ltd	Carthamus tinctorius L., Panax notoginseng F.H.Chen, Eucommia ulmoides Oliv, Panax ginseng C.A.Mey, Citrus reticulata Blanco D.C., Astragalus hamosus L.，Datura metel L., Trionyx sinensis W. and Schizophragma integrifolium Oliv.	Y-prepared according to Pharmacopedia of the People's Republic of China (2000) and National Medical Products Administration Standards:WS-10094 (ZD-0094)-2002-2012Z.

Notes: Concentration of each species in the preparations belong to the core technology of the pharmaceutical companies, so they did not report the grams of each composition.

Supplementary material S4. The details of the search strategy for the primary literatures.

**1. Wang(a) et al. 2023**	**Title (English/Chinese)**	Wang, Y. W., Li, Z., Jin, Y. M., Gu, H., Zhu, B., and Fan, Y. H. (2023). Observation on the Effect of Henggu Gushangyu Mixture on Postoperative Nonunion of Long Bone Fracture. Medical and Pharmacy of Yunnan. 44(2), 33-37.<br>恒古骨伤愈合剂对长骨干骨折术后骨不连的疗效观察
	**URL Link**	https://d.wanfangdata.com.cn/periodical/ChlQZXJpb2RpY2FsQ0hJTmV3UzIwMjMxMjI2Eg15bnl5MjAyMzAyMDEwGghydndwa3ljZw%3D%3D
	**Database**	Wanfang data https://www.wanfangdata.com.cn/
	**Article Level**	It was included in the China Science Citation Database and the CA Chemical Abstracts (USA).
**2. Wang(b) et al. 2023**	**Title (English/Chinese)**	Wang, H. X., Wen, Q., Liu, L., and Meng, D. F. (2023). Clinical study of Henggu Gushang Yuheji combined with Ossotide for injection In treatment of tibial plateau fracture. Drugs & Clinic. 38(2), 432-436.<br>恒古骨伤愈合剂联合注射用骨肽治疗胫骨平台骨折的临床研究
	**URL Link**	https://d.wanfangdata.com.cn/periodical/ChlQZXJpb2RpY2FsQ0hJTmV3UzIwMjMxMjI2EhNnd3l5LXp3eWZjMjAyMzAyMDMzGghyenB5cTJlOA%3D%3D
	**Database**	Wanfang data https://www.wanfangdata.com.cn/
	**Article Level**	It was included in the Chinese Science and Technology Core Journals and the CA Chemical Abstracts (USA).
**3. Hao et al. 2022**	**Title (English/Chinese)**	Hao, M., Liu, X. H., Yao, X. W., Pan, S., Cao, R., and Cao, J. H. (2022). Effects of Osteoking on Bone Metabolism and Bone Mineral Density After Surgery of Osteoporotic Vertebral Compression Fractures. Evaluation and Analysis of Drug-Use in Hospitals of China. 22(6), 664-667<br>恒古骨伤愈合剂对骨质疏松性椎体压缩性骨折术后骨代谢和骨密度的影响
	**URL Link**	https://d.wanfangdata.com.cn/periodical/ChlQZXJpb2RpY2FsQ0hJTmV3UzIwMjMxMjI2EhR6Z3l5eXlwanlmeDIwMjIwNjAwNxoIcWNpeTU4d2s%3D
	**Database**	Wanfang data https://www.wanfangdata.com.cn/
	**Article Level**	It was included in the Chinese Science and Technology Core Journals, the World Journal Clout Index (WJCI), and the Japan Science and Technology Agency (JST).
**4. He et al. 2022**	**Title (English/Chinese)**	He, P. L., Yue, C., Chen, J. M., Ma, M. X., Yang, G. Y., Wang, Q. Y., and Liu, Y. W. (2022). Observation on the curative effect of Henggu Gushangyu Mixture in the treatment of femoral intertrochanteric fracture after internal fixation. Fujian Journal of TCM. 53(5), 60-62<br>恒古骨伤愈合剂治疗股骨转子间骨折内固定术后疗效观察
	**URL Link**	https://d.wanfangdata.com.cn/periodical/ChlQZXJpb2RpY2FsQ0hJTmV3UzIwMjMxMjI2Eg5manp5eTIwMjIwNTAxORoIeWhxa2JkaWM%3D
	**Database**	Wanfang data https://www.wanfangdata.com.cn/

	**Article Level**	It was included in the China Academic Journals (CD-ROM) database, which is maintained by Tsinghua University in China.
**5. Li et al. 2021**	**Title (English/Chinese)**	Li, C. Y., Wu, G. Z., Chen, R., Zhang, W., Zhang, P. J., and Tang, J. (2021). Effect of Osteoking combined with closed reduction and percutaneous locking plate fixation on postoperative healing of tibial fracture. China Medical Herald. 18(33), 94-98.<br>恒古骨伤愈合剂联合闭合复位经皮锁定钢板固定对胫骨骨折术后愈合的影响
	**URL Link**	https://d.wanfangdata.com.cn/periodical/ChlQZXJpb2RpY2FsQ0hJTmV3UzIwMjMxMjI2Eg95eWN5engyMDIxMzMwMjIaCDNjenRpdmQ1
	**Database**	Wanfang data https://www.wanfangdata.com.cn/
	**Article Level**	It was included in the Chinese Science and Technology Core Journals, the CA Chemical Abstracts (USA), and the Japan Science and Technology Agency (JST).
**6. He et al. 2020**	**Title (English/Chinese)**	He, B. J., Mao, Q., Hua, J., and Tong, P. J. (2020). Application of Osteoking to patients who received surgery for treatment of middle — lower tibial fractures and its mechanism of action. J Trad Chin Orthop Trauma. 32(5), 15-21.<br>恒古骨伤愈合剂在胫骨中下段骨折术后的应用及作用机制
	**URL Link**	https://qikan.cqvip.com/Qikan/Article/Detail?id=7101914889&from=Qikan_Search_Index
	**Database**	Wanfang data https://www.wanfangdata.com.cn/
	**Article Level**	It was included in the Chinese Science and Technology Core Journals and the Japan Science and Technology Agency (JST).
**7. Han et al. 2021**	**Title (English/Chinese)**	Han, Y. F., and Liu, Y. W. (2021). Clinical Analysis of Henggu Gushangyu Mixture in the Treatment of Femoral Neck Fracture. World Latest Medicine Information. 21(35), 211-212.<br>恒古骨伤愈合剂治疗股骨颈骨折的临床疗效分析
	**URL Link**	https://d.wanfangdata.com.cn/periodical/ChlQZXJpb2RpY2FsQ0hJTmV3UzIwMjMxMjI2EhpRS0JKQkQyMDIxMjAyMTA4MTMwMDAwMjQ5ORoIZGZ6ZzJyN3E%3D
	**Database**	Wanfang data https://www.wanfangdata.com.cn/
	**Article Level**	It was included in the electronic journals approved by the General Administration of Press and Publication of the People's Republic of China.
**8. Shen 2015**	**Title (English/Chinese)**	Shen, L. (2015). Clinical Study on Traumatic Rib Fracture Treated with Henggu Gushangyu Mixture. Kunming medical university.<br>恒古骨伤愈合剂治疗创伤性肋骨骨折的临床研究
	**URL Link**	https://www.cnki.net/KCMS/detail/detail.aspx?dbcode=CMFD&dbname=CMFD201601&filename=1015634472.nh&uniplatform=OVERSEA&v=L5YXsd3kfkZeoYpnd9d5JgytK1sPOyfRx_M3cuwYlxXxZfAJMEicbC5d72DtJKP8
	**Database**	China National Knowledge Infrastructure (CNKI) https://www.cnki.net/

	**Article Level**	It was included in the China National Knowledge Infrastructure.
**9. Zhu**<br><br>**2014**	**Title**<br><br>**(English/Chinese)**	Zhu, Z. Y. (2014). Clinical effect analysis of open reduction Judet bone grafting combined with Henggu Gushangyu mixture and traditional Chinese medicine hot compress therapy in the treatment of nonunion of femoral shaft fracture. Shandong university of traditional chinese medicine<br>切开复位Judet植骨术配合恒古骨伤愈合剂及中药热敷疗法治疗股骨干骨折不愈合的临床效果分析
	**URL Link**	https://www.cnki.net/KCMS/detail/detail.aspx?dbcode=CMFD&dbname=CMFD201501&filename=1015511837.nh&uniplatform=OVERSEA&v=UbfEYPGPU_8ADvqnMQ9xGirRjhzD00WUsPwLCrZAnlteSCsKokea9jlTIurOW6m
	**Database**	China National Knowledge Infrastructure (CNKI) https://www.cnki.net/
	**Article Level**	It was included in the China National Knowledge Infrastructure.
**10. Zhang et al.**<br><br>**2013**	**Title**<br><br>**(English/Chinese)**	Zhang, Z. B., Wang, P., Luo, H. L., and Liu, T. (2013). Operation combined with traditional Chinese medicine treatment of distal radius comminuted fracture and intra-articular fracture curative effect observation. Asia-Pacific Traditional Medicine. 9(6), 115-116.<br>手术结合中药治疗桡骨远端粉碎性骨折及关节内骨折疗效观察
	**URL Link**	https://d.wanfangdata.com.cn/periodical/ChlQZXJpb2RpY2FsQ0hJTmV3UzIwMjMxMjI2Eg95dGN0eXkyMDEzMDYwNTcaCDdvc2xiZ3Zu
	**Database**	Wanfang data https://www.wanfangdata.com.cn/
	**Article Level**	It was included in the China National Knowledge Infrastructure and the Japan Science and Technology Agency (JST).
**11. Luo et al.**<br><br>**2011**	**Title**<br><br>**(English/Chinese)**	Luo, D. J., Zhao, H. B., Zhou, X., Dong, X. L., Li, L. Z., Wang, W. Z., and Xiong, H. (2011). Efficacy evaluation of salmon calcitonin combined with Henggu Gushangyu Mixture in the treatment of lumbar OPF. Chin J Endocr Surg. 5(3), 158-160.<br>鲑鱼降钙素联合恒古骨伤愈合剂治疗腰椎OPF疗效分析
	**URL Link**	https://rs.yiigle.com/CN115399202004/190764.htm
	**Database**	Wanfang data https://www.wanfangdata.com.cn/
	**Article Level**	It was included in the Chinese core journal criterion for pku, the Chinese Science and Technology Core Journals, and the CA Chemical Abstracts (USA).
**12. Hu et al.**<br><br>**2005**	**Title**<br><br>**(English/Chinese)**	Hu, M., Zhao, H. B., Wang, B., Liang, H. S., Zhang, C. Q., Zheng, H. Y., and Zhao, X. L. (2005). Clinical Observation on Promoting Postoperative Healing of Lower Tibial Fractures by Henggu Gushangyu Mixture. CJITWM. 25(2), 160-161<br>恒古骨伤愈合剂促进胫骨下段骨折术后愈合临床观察
	**URL Link**	https://d.wanfangdata.com.cn/periodical/ChlQZXJpb2RpY2FsQ0hJTmV3UzIwMjMxMjI2Eg56eHlqaDIwMDUwMjAxNxoIajg3NXE2ZGk%3D
	**Database**	Wanfang data https://www.wanfangdata.com.cn/

	**Article Level**	It was included in the Chinese core journal criterion for pku, the Chinese Science Citation Database (CSCD), the Chinese Science and Technology Core Journals, the CA Chemical Abstracts (USA), the World Journal Clout Index (WJCI), and the Japan Science and Technology Agency (JST).
**13. Min 2023**	**Title (English/Chinese)**	Min, R. (2023). Analysis of Therapeutic Effect of Henggu Gushang Healing Agent Combined with PFNA on Intertrochanteric Fracture of Femur. JOURNAL OF JIANGXI UNIVERSITY OF CM. 35(4), 43-46.<br>PFNA术后联合恒古骨伤愈合剂对股骨粗隆间骨折的疗效观察
	**URL Link**	https://d.wanfangdata.com.cn/periodical/ChlQZXJpb2RpY2FsQ0hJTmV3UzIwMjMxMjI2EhFqeHp5eHl4YjIwMjMwNDAxMhoIbDRtb2lybmQ%3D
	**Database**	Wanfang data https://www.wanfangdata.com.cn/
	**Article Level**	It was included in the China National Knowledge Infrastructure.

Supplementary Figure 1.

Study or Subgroup	Experimental Mean	Experimental SD	Experimental Total	Control Mean	Control SD	Control Total	Weight	Std. Mean Difference IV, Random, 95% CI
**1.1.1 Limbs long backbone**								
He 2020	22.47	3.32	32	26.79	2.89	33	9.1%	-1.37 [-1.92, -0.83]
Hu 2005	38	7.3	72	51	7.2	54	9.6%	-1.78 [-2.20, -1.36]
Li 2021	3.81	0.82	71	4.74	0.74	69	9.8%	-1.18 [-1.54, -0.82]
Wang(a) 2023	21.25	2.7	28	30.19	2.88	28	8.1%	-3.16 [-3.96, -2.36]
Wang(b) 2023	10.97	2.21	71	13.12	2.41	72	9.8%	-0.92 [-1.27, -0.58]
Zhang 2013	21.6	4.2	63	32.5	5.1	63	9.5%	-2.32 [-2.77, -1.86]
Zhu 2014	3.8	0.92	12	4.1	0.86	12	8.0%	-0.33 [-1.13, 0.48]
**Subtotal (95% CI)**			**349**			**331**	**63.8%**	**-1.57 [-2.10, -1.03]**
Heterogeneity: Tau² = 0.45; Chi² = 52.43, df = 6 (P < 0.00001); I² = 89%								
Test for overall effect: Z = 5.73 (P < 0.00001)								
**1.1.2 Vertebrae**								
Hao 2022	82.52	8.21	33	99.09	11.06	33	9.0%	-1.68 [-2.25, -1.12]
**Subtotal (95% CI)**			**33**			**33**	**9.0%**	**-1.68 [-2.25, -1.12]**
Heterogeneity: Not applicable								
Test for overall effect: Z = 5.82 (P < 0.00001)								
**1.1.3 Hip**								
He 2022	21.79	3.44	35	27.09	3.26	35	9.1%	-1.56 [-2.10, -1.02]
Min 2023	25.53	3.79	36	27.44	2.68	34	9.4%	-0.57 [-1.05, -0.09]
**Subtotal (95% CI)**			**71**			**69**	**18.5%**	**-1.06 [-2.03, -0.09]**
Heterogeneity: Tau² = 0.42; Chi² = 7.26, df = 1 (P = 0.007); I² = 86%								
Test for overall effect: Z = 2.14 (P = 0.03)								
**1.1.4 Rib**								
Shen 2015	8.18	0.94	46	12.57	1.46	46	8.6%	-3.55 [-4.21, -2.88]
**Subtotal (95% CI)**			**46**			**46**	**8.6%**	**-3.55 [-4.21, -2.88]**
Heterogeneity: Not applicable								
Test for overall effect: Z = 10.46 (P < 0.00001)								
**Total (95% CI)**			**499**			**479**	**100.0%**	**-1.66 [-2.14, -1.17]**
Heterogeneity: Tau² = 0.60; Chi² = 103.93, df = 10 (P < 0.00001); I² = 90%								
Test for overall effect: Z = 6.68 (P < 0.00001)								
Test for subgroup differences: Chi² = 28.12, df = 3 (P < 0.00001), I² = 89.3%								

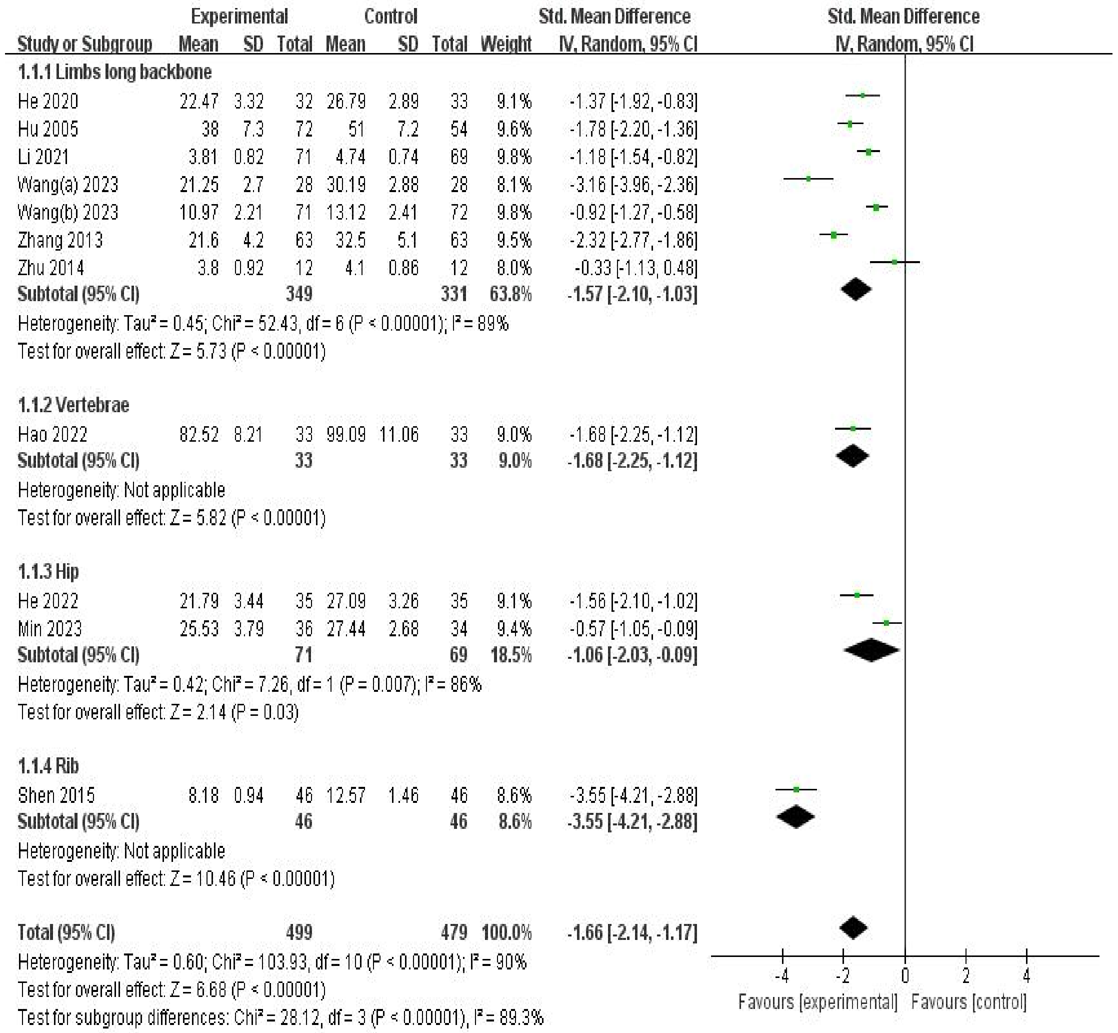

Supplementary Figure 2.

Study or Subgroup	Experimental Mean	Experimental SD	Experimental Total	Control Mean	Control SD	Control Total	Weight	Std. Mean Difference IV, Random, 95% CI
**2.1.1 OK+BT vs BT**								
Hao 2022	82.52	8.21	33	99.09	11.06	33	9.0%	-1.68 [-2.25, -1.12]
Li 2021	3.81	0.82	71	4.74	0.74	69	9.8%	-1.18 [-1.54, -0.82]
Shen 2015	8.18	0.94	46	12.57	1.46	46	8.6%	-3.55 [-4.21, -2.88]
Wang(a) 2023	21.25	2.7	28	30.19	2.88	28	8.1%	-3.16 [-3.96, -2.36]
Wang(b) 2023	10.97	2.21	71	13.12	2.41	72	9.8%	-0.92 [-1.27, -0.58]
Zhang 2013	21.6	4.2	63	32.5	5.1	63	9.5%	-2.32 [-2.77, -1.86]
Zhu 2014	3.8	0.92	12	4.1	0.86	12	8.0%	-0.33 [-1.13, 0.48]
**Subtotal (95% CI)**			**324**			**323**	**62.8%**	**-1.86 [-2.60, -1.13]**
Heterogeneity: Tau² = 0.89; Chi² = 86.59, df = 6 (P < 0.00001); I² = 93%								
Test for overall effect: Z = 4.97 (P < 0.00001)								
**2.1.2 OK vs OCMT**								
He 2020	22.47	3.32	32	26.79	2.89	33	9.1%	-1.37 [-1.92, -0.83]
He 2022	21.79	3.44	35	27.09	3.26	35	9.1%	-1.56 [-2.10, -1.02]
Hu 2005	38	7.3	72	51	7.2	54	9.6%	-1.78 [-2.20, -1.36]
Min 2023	25.53	3.79	36	27.44	2.68	34	9.4%	-0.57 [-1.05, -0.09]
**Subtotal (95% CI)**			**175**			**156**	**37.2%**	**-1.32 [-1.87, -0.78]**
Heterogeneity: Tau² = 0.25; Chi² = 14.82, df = 3 (P = 0.002); I² = 80%								
Test for overall effect: Z = 4.75 (P < 0.00001)								
**Total (95% CI)**			**499**			**479**	**100.0%**	**-1.66 [-2.14, -1.17]**
Heterogeneity: Tau² = 0.60; Chi² = 103.93, df = 10 (P < 0.00001); I² = 90%								
Test for overall effect: Z = 6.68 (P < 0.00001)								
Test for subgroup differences: Chi² = 1.32, df = 1 (P = 0.25), I² = 24.5%								

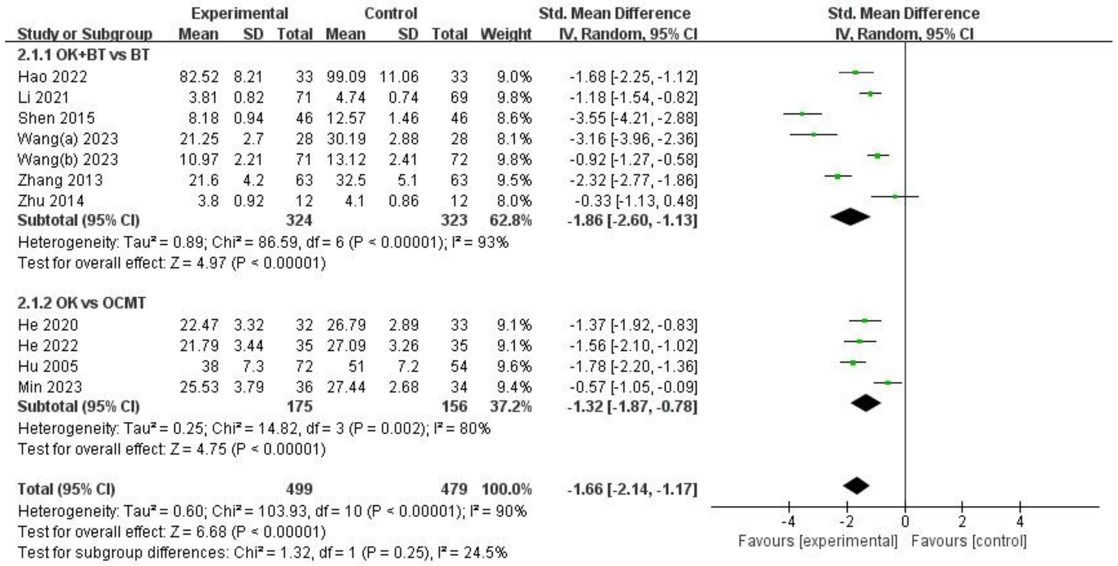

Supplementary Figure 3.

Study or Subgroup	Experimental Mean	Experimental SD	Experimental Total	Control Mean	Control SD	Control Total	Weight	Std. Mean Difference IV, Random, 95% CI
**3.1.1 ≤4 weeks**								
He 2020	22.47	3.32	32	26.79	2.89	33	9.1%	-1.37 [-1.92, -0.83]
He 2022	21.79	3.44	35	27.09	3.26	35	9.1%	-1.56 [-2.10, -1.02]
Hu 2005	38	7.3	72	51	7.2	54	9.6%	-1.78 [-2.20, -1.36]
Li 2021	3.81	0.82	71	4.74	0.74	69	9.8%	-1.18 [-1.54, -0.82]
Min 2023	25.53	3.79	36	27.44	2.68	34	9.4%	-0.57 [-1.05, -0.09]
Shen 2015	8.18	0.94	46	12.57	1.46	46	8.6%	-3.55 [-4.21, -2.88]
Wang(b) 2023	10.97	2.21	71	13.12	2.41	72	9.8%	-0.92 [-1.27, -0.58]
**Subtotal (95% CI)**			**363**			**343**	**65.4%**	**-1.53 [-2.09, -0.97]**
Heterogeneity: Tau² = 0.51; Chi² = 63.44, df = 6 (P < 0.00001); I² = 91%								
Test for overall effect: Z = 5.37 (P < 0.00001)								
**3.1.2 >4 weeks**								
Hao 2022	82.52	8.21	33	99.09	11.06	33	9.0%	-1.68 [-2.25, -1.12]
Wang(a) 2023	21.25	2.7	28	30.19	2.88	28	8.1%	-3.16 [-3.96, -2.36]
Zhang 2013	21.6	4.2	63	32.5	5.1	63	9.5%	-2.32 [-2.77, -1.86]
Zhu 2014	3.8	0.92	12	4.1	0.86	12	8.0%	-0.33 [-1.13, 0.48]
**Subtotal (95% CI)**			**136**			**136**	**34.6%**	**-1.88 [-2.84, -0.92]**
Heterogeneity: Tau² = 0.84; Chi² = 27.68, df = 3 (P < 0.00001); I² = 89%								
Test for overall effect: Z = 3.84 (P = 0.0001)								
**Total (95% CI)**			**499**			**479**	**100.0%**	**-1.66 [-2.14, -1.17]**
Heterogeneity: Tau² = 0.60; Chi² = 103.93, df = 10 (P < 0.00001); I² = 90%								
Test for overall effect: Z = 6.68 (P < 0.00001)								
Test for subgroup differences: Chi² = 0.38, df = 1 (P = 0.54), I² = 0%								

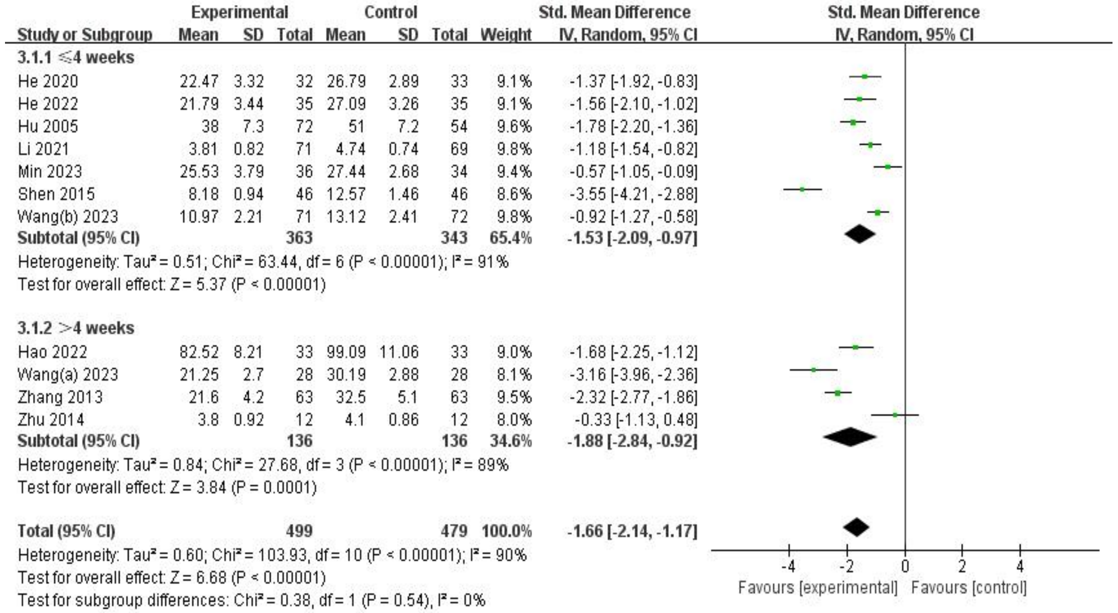

Supplementary Figure 4.

Study or Subgroup	Experimental Events	Experimental Total	Control Events	Control Total	Weight	Odds Ratio M-H, Fixed, 95% CI
**4.1.1 Limbs long backbone**						
He 2020	30	32	25	33	8.7%	4.80 [0.93, 24.69]
Hu 2005	70	72	40	54	7.2%	12.25 [2.65, 56.67]
Li 2021	70	71	60	69	4.9%	10.50 [1.29, 85.28]
Wang(a) 2023	28	28	27	28	2.7%	3.11 [0.12, 79.64]
Wang(b) 2023	64	71	55	72	30.6%	2.83 [1.09, 7.32]
Zhang 2013	53	63	40	63	36.1%	3.05 [1.30, 7.12]
Zhu 2014	11	12	10	12	4.7%	2.20 [0.17, 28.14]
**Subtotal (95% CI)**		**349**		**331**	**95.0%**	**4.18 [2.53, 6.91]**
Total events	326		257			
Heterogeneity: Chi² = 4.12, df = 6 (P = 0.66); I² = 0%						
Test for overall effect: Z = 5.58 (P < 0.00001)						
**4.1.2 Vertebrae**						
Hao 2022	33	33	29	33	2.5%	10.22 [0.53, 197.89]
**Subtotal (95% CI)**		**33**		**33**	**2.5%**	**10.22 [0.53, 197.89]**
Total events	33		29			
Heterogeneity: Not applicable						
Test for overall effect: Z = 1.54 (P = 0.12)						
**4.1.3 Rib**						
Shen 2015	12	12	11	12	2.5%	3.26 [0.12, 88.35]
**Subtotal (95% CI)**		**12**		**12**	**2.5%**	**3.26 [0.12, 88.35]**
Total events	12		11			
Heterogeneity: Not applicable						
Test for overall effect: Z = 0.70 (P = 0.48)						
**Total (95% CI)**		**394**		**376**	**100.0%**	**4.30 [2.64, 7.02]**
Total events	371		297			
Heterogeneity: Chi² = 4.55, df = 8 (P = 0.80); I² = 0%						
Test for overall effect: Z = 5.85 (P < 0.00001)						
Test for subgroup differences: Chi² = 0.37, df = 2 (P = 0.83), I² = 0%						

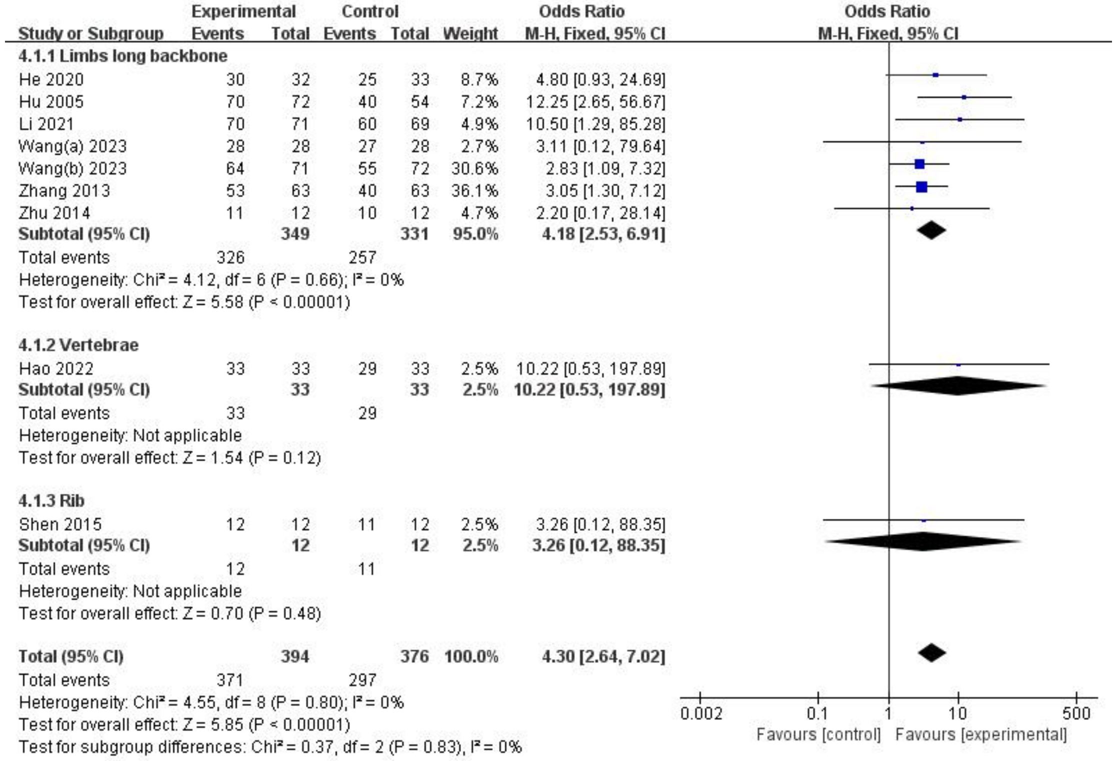

Supplementary Figure 5.

Study or Subgroup	Experimental Events	Experimental Total	Control Events	Control Total	Weight	Odds Ratio M-H, Fixed, 95% CI
**5.1.1 OK+BT vs BT**						
Hao 2022	33	33	29	33	2.5%	10.22 [0.53, 197.89]
Li 2021	70	71	60	69	4.9%	10.50 [1.29, 85.28]
Shen 2015	12	12	11	12	2.5%	3.26 [0.12, 88.35]
Wang(a) 2023	28	28	27	28	2.7%	3.11 [0.12, 79.64]
Wang(b) 2023	64	71	55	72	30.6%	2.83 [1.09, 7.32]
Zhang 2013	53	63	40	63	36.1%	3.05 [1.30, 7.12]
Zhu 2014	11	12	10	12	4.7%	2.20 [0.17, 28.14]
**Subtotal (95% CI)**		**290**		**289**	**84.0%**	**3.57 [2.06, 6.20]**
Total events	271		232			
Heterogeneity: Chi² = 2.02, df = 6 (P = 0.92); I² = 0%						
Test for overall effect: Z = 4.52 (P < 0.00001)						
**5.1.2 OK vs OCMT**						
He 2020	30	32	25	33	8.7%	4.80 [0.93, 24.69]
Hu 2005	70	72	40	54	7.2%	12.25 [2.65, 56.67]
**Subtotal (95% CI)**		**104**		**87**	**16.0%**	**8.17 [2.71, 24.66]**
Total events	100		65			
Heterogeneity: Chi² = 0.67, df = 1 (P = 0.41); I² = 0%						
Test for overall effect: Z = 3.73 (P = 0.0002)						
**Total (95% CI)**		**394**		**376**	**100.0%**	**4.30 [2.64, 7.02]**
Total events	371		297			
Heterogeneity: Chi² = 4.55, df = 8 (P = 0.80); I² = 0%						
Test for overall effect: Z = 5.85 (P < 0.00001)						
Test for subgroup differences: Chi² = 1.73, df = 1 (P = 0.19), I² = 42.1%						

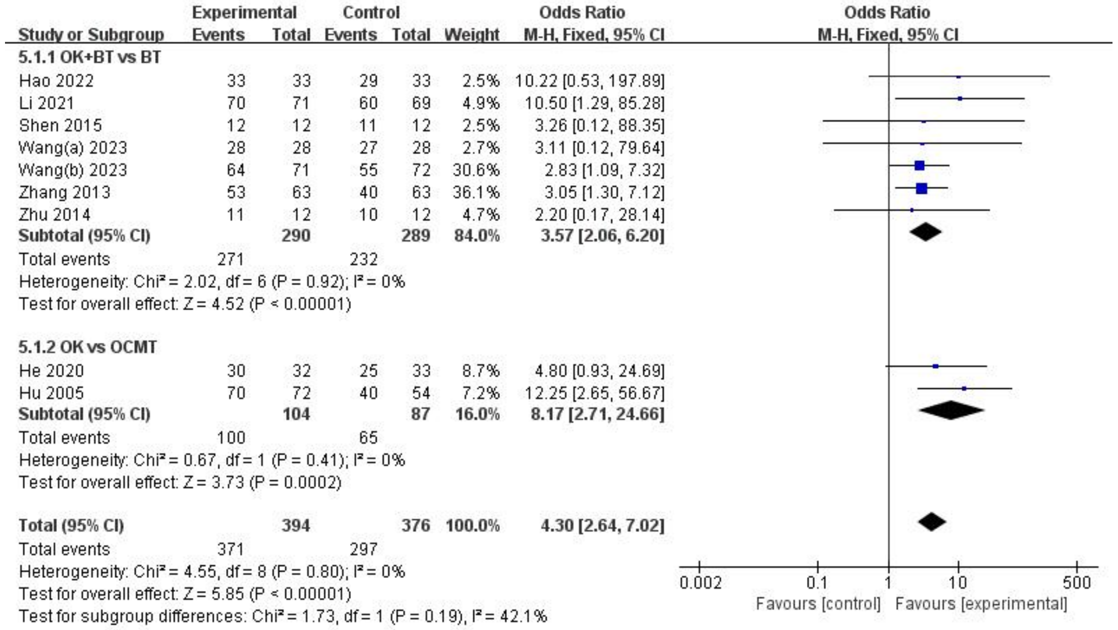

Supplementary Figure 6.

Study or Subgroup	Experimental Events	Experimental Total	Control Events	Control Total	Weight	Odds Ratio M-H, Fixed, 95% CI
**6.1.1 ≤4 weeks**						
He 2020	30	32	25	33	8.7%	4.80 [0.93, 24.69]
Hu 2005	70	72	40	54	7.2%	12.25 [2.65, 56.67]
Li 2021	70	71	60	69	4.9%	10.50 [1.29, 85.28]
Shen 2015	12	12	11	12	2.5%	3.26 [0.12, 88.35]
Wang(b) 2023	64	71	55	72	30.6%	2.83 [1.09, 7.32]
**Subtotal (95% CI)**		**258**		**240**	**54.0%**	**5.12 [2.67, 9.82]**
Total events	246		191			
Heterogeneity: Chi² = 3.28, df = 4 (P = 0.51); I² = 0%						
Test for overall effect: Z = 4.91 (P < 0.00001)						
**6.1.2 >4 weeks**						
Hao 2022	33	33	29	33	2.5%	10.22 [0.53, 197.89]
Wang(a) 2023	28	28	27	28	2.7%	3.11 [0.12, 79.64]
Zhang 2013	53	63	40	63	36.1%	3.05 [1.30, 7.12]
Zhu 2014	11	12	10	12	4.7%	2.20 [0.17, 28.14]
**Subtotal (95% CI)**		**136**		**136**	**46.0%**	**3.35 [1.59, 7.07]**
Total events	125		106			
Heterogeneity: Chi² = 0.70, df = 3 (P = 0.87); I² = 0%						
Test for overall effect: Z = 3.17 (P = 0.002)						
**Total (95% CI)**		**394**		**376**	**100.0%**	**4.30 [2.64, 7.02]**
Total events	371		297			
Heterogeneity: Chi² = 4.55, df = 8 (P = 0.80); I² = 0%						
Test for overall effect: Z = 5.85 (P < 0.00001)						
Test for subgroup differences: Chi² = 0.70, df = 1 (P = 0.40), I² = 0%						

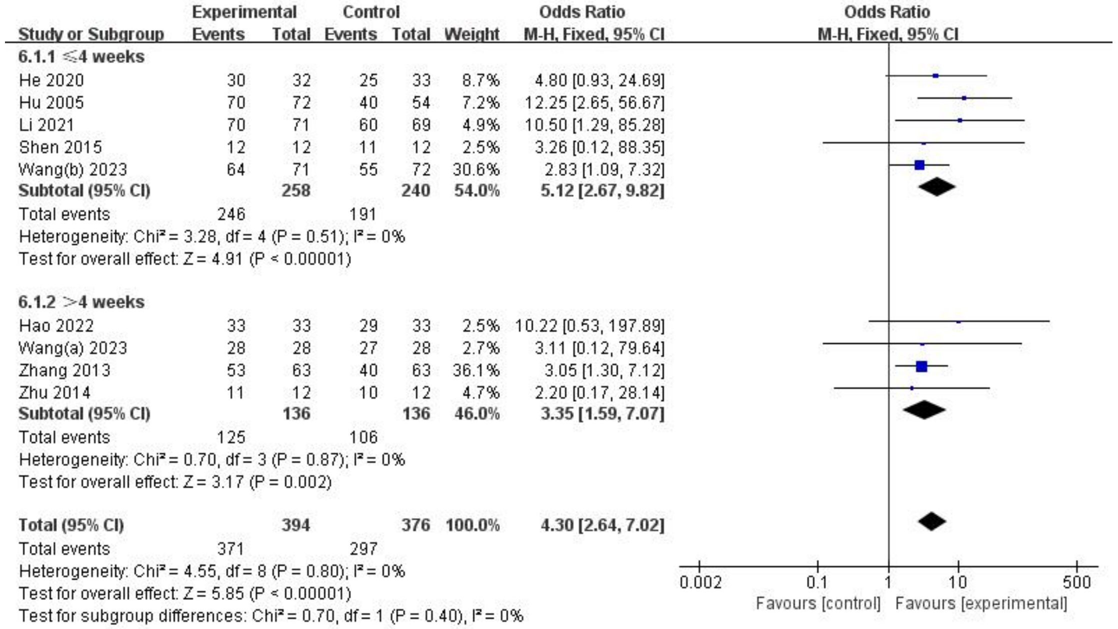

Supplementary Figure 7.

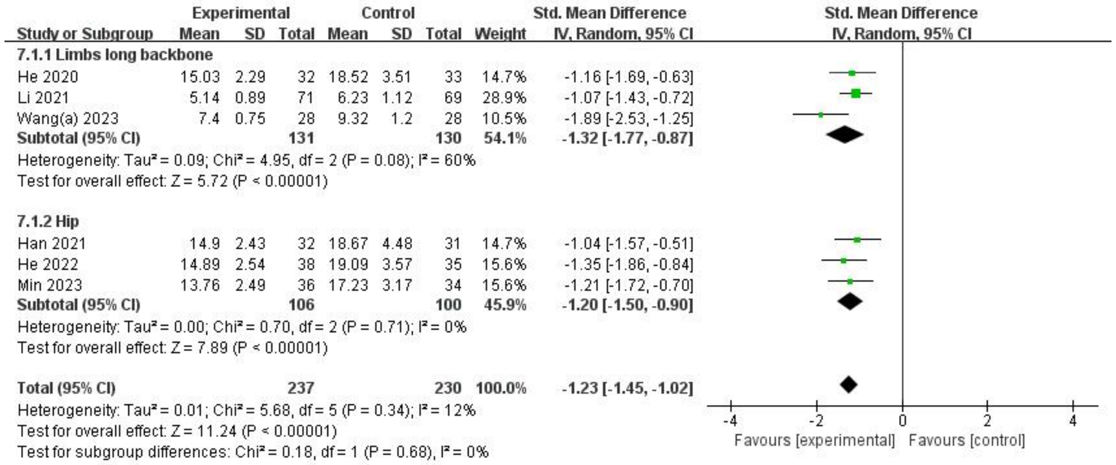

Supplementary Figure 8.

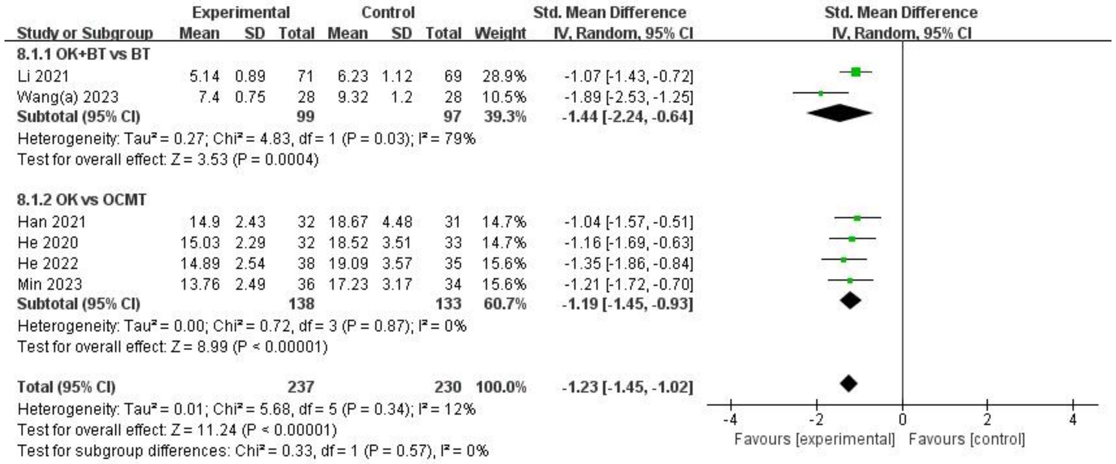

Supplementary Figure 9.

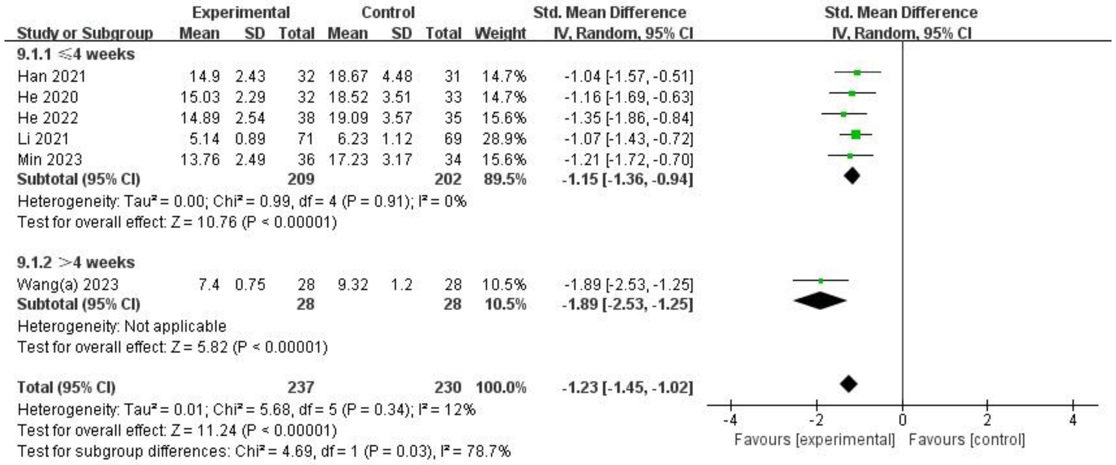

Supplementary Figure 10.

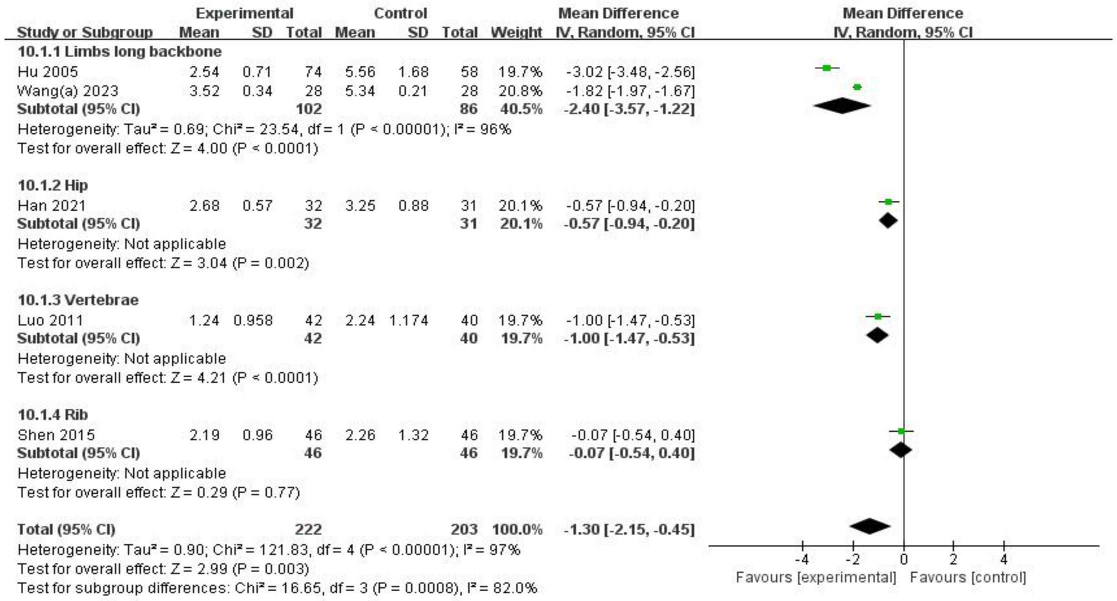

Supplementary Figure 11.

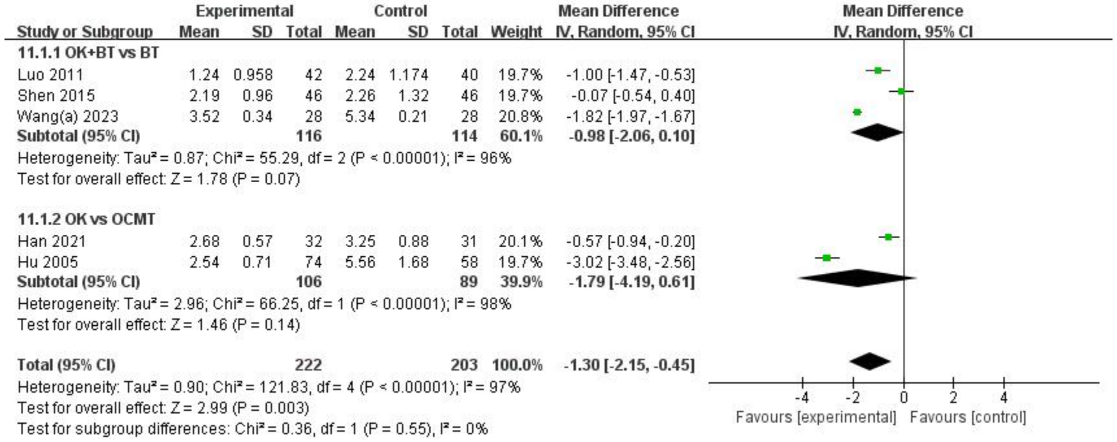

Supplementary Figure 12.

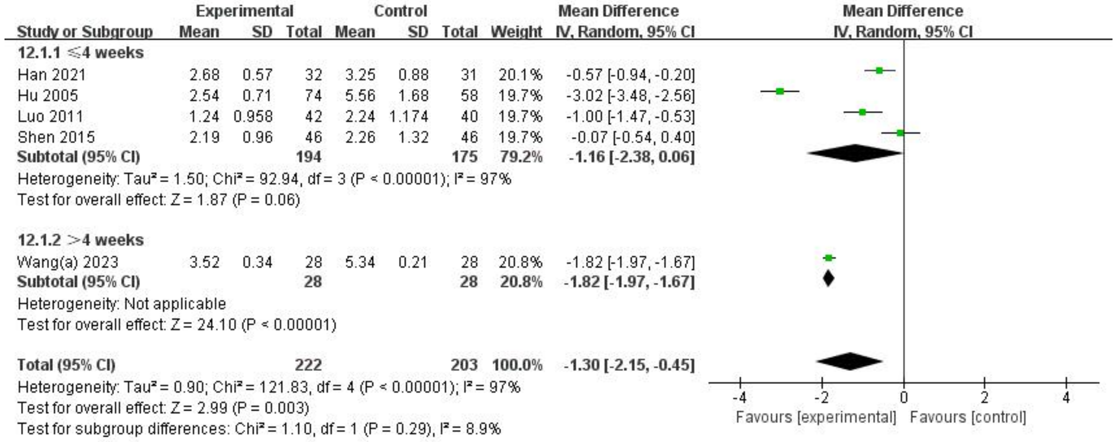

Supplementary Figure 13.

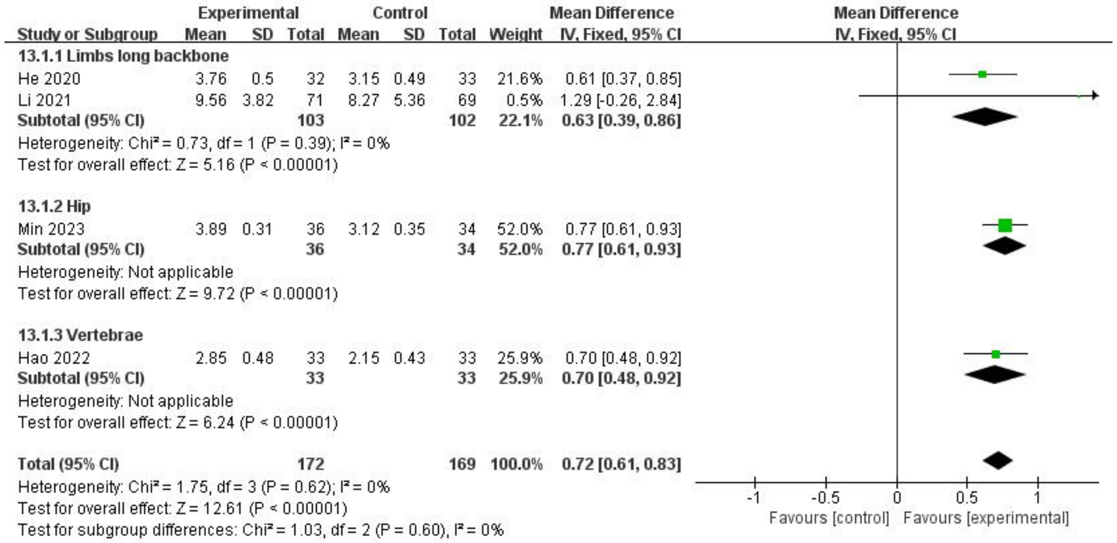

Supplementary Figure 14.

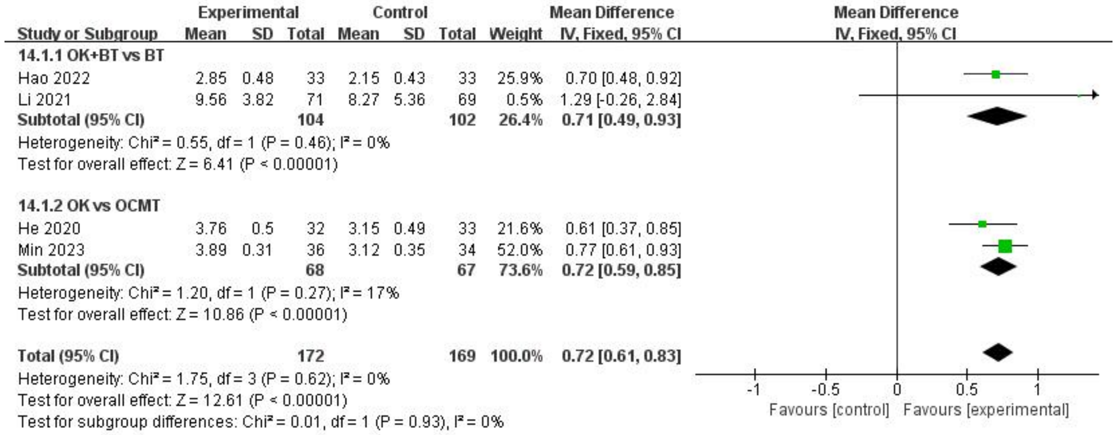

Supplementary Figure 15.

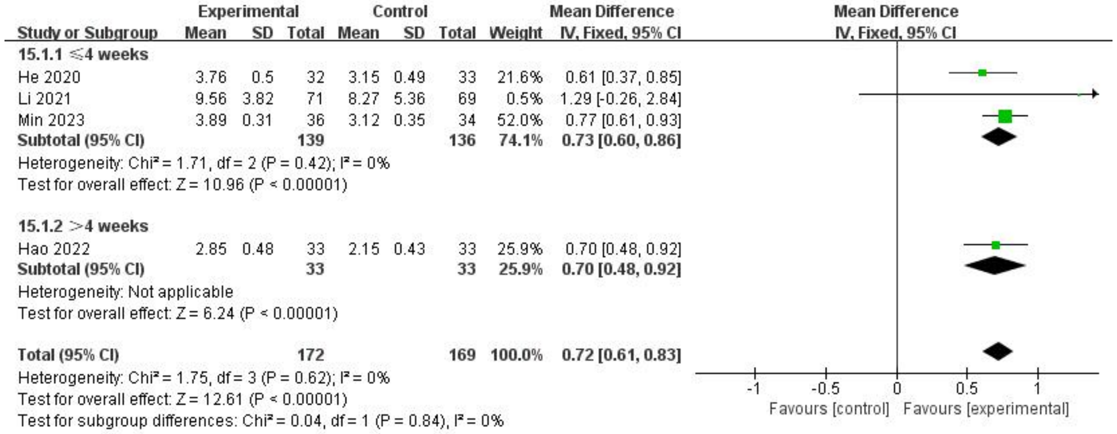

Supplementary Figure 16.

Study or Subgroup	Experimental Mean	Experimental SD	Experimental Total	Control Mean	Control SD	Control Total	Weight	Mean Difference IV, Random, 95% CI
**16.1.1 Limbs long backbone**								
He 2020	98.32	5.47	71	86.35	6.34	69	16.9%	11.97 [10.01, 13.93]
Li 2021	128.91	10.1	32	112.33	7.86	33	16.5%	16.58 [12.17, 20.99]
Zhang 2013	124.36	15.95	63	102.48	9.52	63	16.5%	21.88 [17.29, 26.47]
**Subtotal (95% CI)**			**166**			**165**	**49.9%**	**16.54 [10.54, 22.55]**
Heterogeneity: Tau² = 24.48; Chi² = 16.75, df = 2 (P = 0.0002); I² = 88%								
Test for overall effect: Z = 5.40 (P < 0.00001)								
**16.1.2 Hip**								
Min 2023	125.39	9.18	36	112.33	7.62	34	16.6%	13.06 [9.12, 17.00]
**Subtotal (95% CI)**			**36**			**34**	**16.6%**	**13.06 [9.12, 17.00]**
Heterogeneity: Not applicable								
Test for overall effect: Z = 6.49 (P < 0.00001)								
**16.1.3 Rib**								
Shen 2015	133.06	5.43	46	92.87	8.83	46	16.8%	40.19 [37.19, 43.19]
**Subtotal (95% CI)**			**46**			**46**	**16.8%**	**40.19 [37.19, 43.19]**
Heterogeneity: Not applicable								
Test for overall effect: Z = 26.30 (P < 0.00001)								
**16.1.4 Vertebrae**								
Hao 2022	96.3	7.1	33	89.81	6.3	33	16.7%	6.49 [3.25, 9.73]
**Subtotal (95% CI)**			**33**			**33**	**16.7%**	**6.49 [3.25, 9.73]**
Heterogeneity: Not applicable								
Test for overall effect: Z = 3.93 (P < 0.0001)								
**Total (95% CI)**			**281**			**278**	**100.0%**	**18.36 [8.00, 28.72]**
Heterogeneity: Tau² = 164.08; Chi² = 303.76, df = 5 (P < 0.00001); I² = 98%								
Test for overall effect: Z = 3.47 (P = 0.0005)								
Test for subgroup differences: Chi² = 252.26, df = 3 (P < 0.00001), I² = 98.8%								

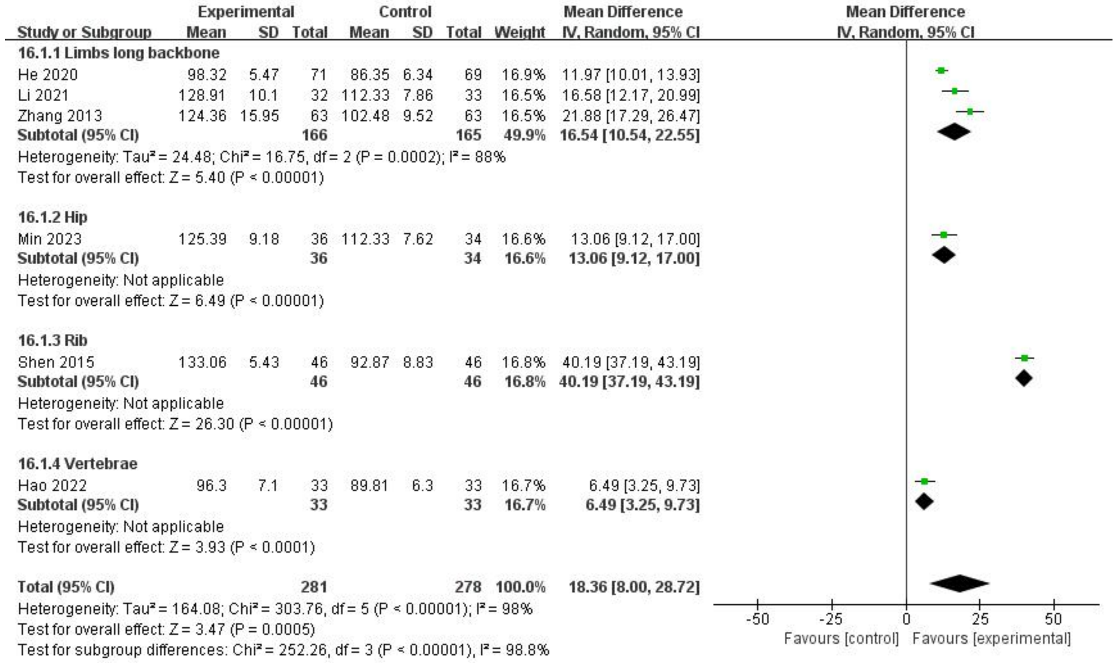

Supplementary Figure 17.

Study or Subgroup	Experimental Mean	Experimental SD	Experimental Total	Control Mean	Control SD	Control Total	Weight	Mean Difference IV, Random, 95% CI
**17.1.1 OK+BT vs BT**								
Hao 2022	96.3	7.1	33	89.81	6.3	33	16.7%	6.49 [3.25, 9.73]
Li 2021	128.91	10.1	32	112.33	7.86	33	16.5%	16.58 [12.17, 20.99]
Shen 2015	133.06	5.43	46	92.87	8.83	46	16.8%	40.19 [37.19, 43.19]
Zhang 2013	124.36	15.95	63	102.48	9.52	63	16.5%	21.88 [17.29, 26.47]
**Subtotal (95% CI)**			**174**			**175**	**66.5%**	**21.30 [4.94, 37.66]**
Heterogeneity: Tau² = 274.93; Chi² = 234.69, df = 3 (P < 0.00001); I² = 99%								
Test for overall effect: Z = 2.55 (P = 0.01)								
**17.1.2 OK vs OCMT**								
He 2020	98.32	5.47	71	86.35	6.34	69	16.9%	11.97 [10.01, 13.93]
Min 2023	125.39	9.18	36	112.33	7.62	34	16.6%	13.06 [9.12, 17.00]
**Subtotal (95% CI)**			**107**			**103**	**33.5%**	**12.19 [10.43, 13.94]**
Heterogeneity: Tau² = 0.00; Chi² = 0.24, df = 1 (P = 0.63); I² = 0%								
Test for overall effect: Z = 13.59 (P < 0.00001)								
**Total (95% CI)**			**281**			**278**	**100.0%**	**18.36 [8.00, 28.72]**
Heterogeneity: Tau² = 164.08; Chi² = 303.76, df = 5 (P < 0.00001); I² = 98%								
Test for overall effect: Z = 3.47 (P = 0.0005)								
Test for subgroup differences: Chi² = 1.18, df = 1 (P = 0.28), I² = 15.1%								

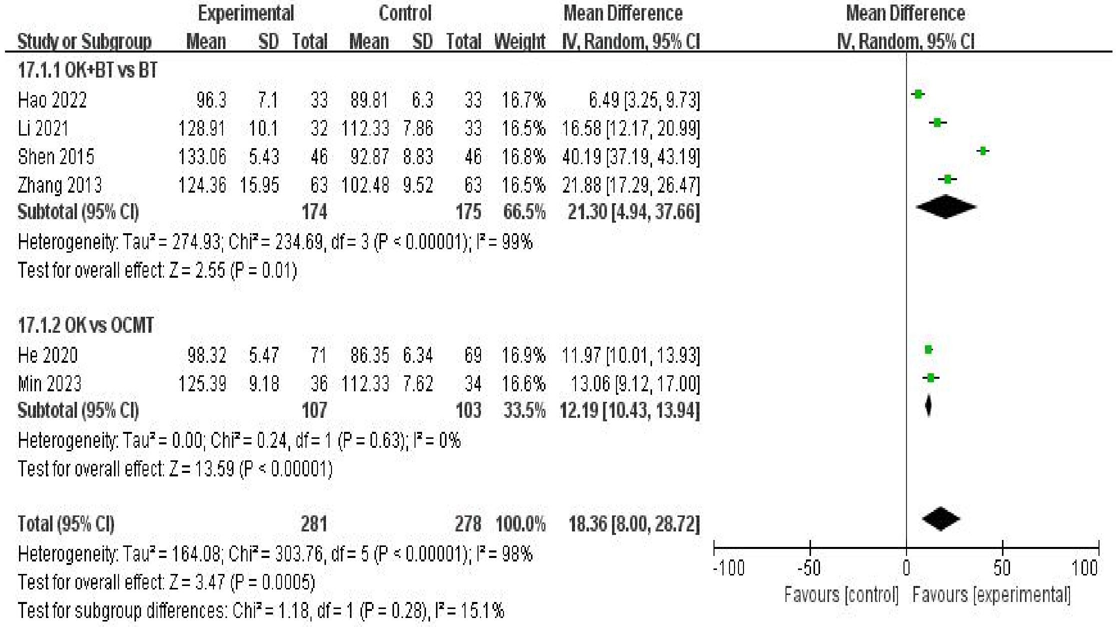

Supplementary Figure 18.

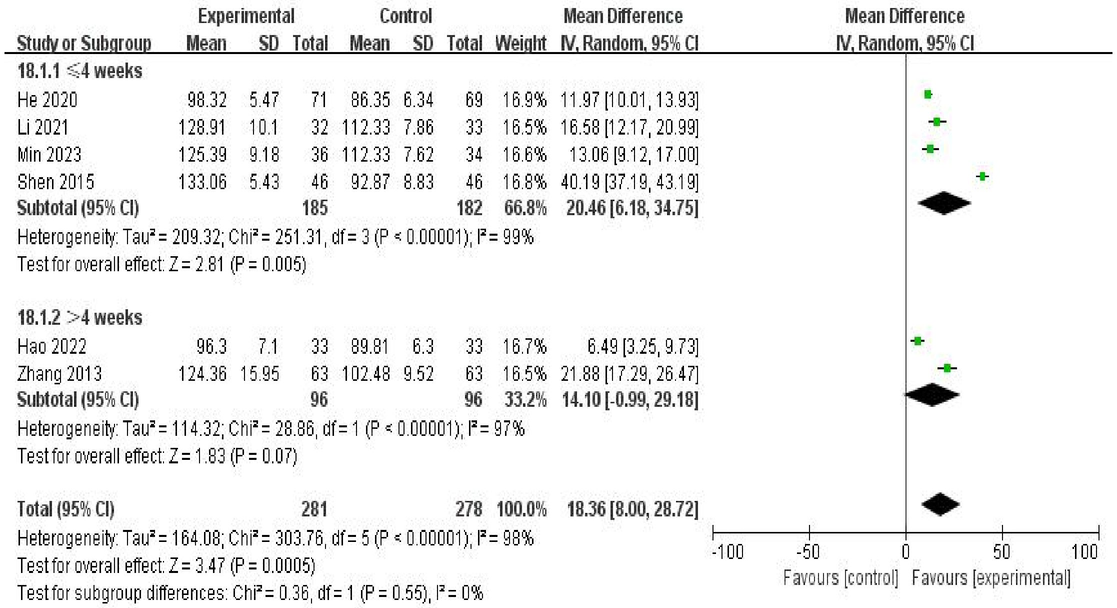

Supplementary Table 1. Sensitivity analysis for FHT, FHR.

Outcomes	References	Effect size	95% CI	*P*	I^2
FHT	Hao 2022	-1.66	-2.19, -1.13	< 0.00001	91%
	He 2020	-1.69	-2.22, -1.15	< 0.00001	91%
	He 2022	-1.67	-2.20, -1.13	< 0.00001	91%
	Hu 2005	-1.65	-2.19, -1.10	< 0.00001	91%
	Li 2021	-1.71	-2.26, -1.16	< 0.00001	91%
	Min 2023	-1.77	-2.27, -1.27	< 0.00001	90%
	Shen 2015	-1.47	-1.89, -1.05	< 0.00001	86%
	Wang(a) 2023	-1.52	-2.00, -1.05	< 0.00001	90%
	Wang(b) 2023	-1.74	-2.27, -1.21	< 0.00001	90%
	Zhang 2013	-1.59	-2.09, -1.08	< 0.00001	90%

	Zhu 2014	-1.77	-2.27, -1.27	< 0.00001	91%
FHR	Hao 2022	4.16	2.53, 6.83	< 0.00001	0%
	He 2020	4.26	2.55, 7.11	< 0.00001	0%
	Hu 2005	3.69	2.19, 6.21	< 0.00001	0%
	Li 2021	3.99	2.41, 6.61	< 0.00001	0%
	Shen 2015	4.33	2.64, 7.10	< 0.00001	0%
	Wang(a) 2023	4.34	2.65, 7.11	< 0.00001	0%
	Wang(b) 2023	4.96	2.79, 8.80	< 0.00001	0%
	Zhang 2013	5.02	2.74, 9.17	< 0.00001	0%
	Zhu 2014	4.41	2.68, 7.26	< 0.00001	0%
